# Decoding *MexB* efflux pump genes: structural, molecular, and phylogenetic analysis of multidrug-resistant and extensively drug-resistant *Pseudomonas aeruginosa*

**DOI:** 10.3389/fcimb.2024.1519737

**Published:** 2025-01-21

**Authors:** Muhammad Bilal Habib, Naseer Ali Shah, Afreenish Amir, Huda Ahmed Alghamdi, Muhammad Haseeb Tariq, Kiran Nisa, Mariam Ammoun

**Affiliations:** ^1^ Department of Biosciences, COMSATS University, Islamabad, Pakistan; ^2^ Department of Microbiology, National Institute of Health, Islamabad, Pakistan; ^3^ Department of Biology, College of Sciences, King Khalid University, Abha, Saudi Arabia; ^4^ Department of Pathology, Viva Health Laboratories, Windsor, United Kingdom

**Keywords:** *Pseudomonas aeruginosa*, antimicrobial resistance, efflux pump, protein domain, phylogenetic analysis, MolProbity, Ramachandran plot

## Abstract

**Objective:**

Emerging drug resistance in *Pseudomonas aeruginosa* is of great concern in clinical settings. *P. aeruginosa* activates its efflux-pump system in order to evade the effect of antibiotics. The current investigation aims to detect *MexB* genes in *P. aeruginosa*, their structural and molecular analysis and their impact on antimicrobial susceptibility profiling.

**Methods:**

A total of 42 clinical specimens were aseptically collected from hospitalized patients who had underlying infections related to medical implants. Matrix-assisted laser desorption ionization-time of flight (MALDI-ToF) were used for the identification of isolates. The methods used in this study were antibiotic susceptibility profiling, minimum inhibitory concentration (MIC), polymerase chain reaction (PCR), sanger sequencing, phylogenetic analysis, MolProbity score, Ramachandran plot analysis and multiple sequence alignment.

**Results:**

The highest resistance was shown by *P. aeruginosa* against cefoperazone (67%), gentamycin and amikacin (66%) each, followed by cefotaxime (64%). The prevalence of multi-drug resistant (MDR) and extensively drug resistant (XDR) was 57% and 12%, respectively. The presence of an active efflux-pump system was indicated by the *MexB* genes found in most of the resistant isolates (p<0.05). Following addition of efflux pump inhibitor carbonyl cyanide m-chlorophenyl hydrazone (CCCP), a significant decrease (p<0.05) in MIC was observed in resistance, that revealed the presence of active efflux pump system. Phylogenetic analysis revealed evolutionary relationships with the *P. aeruginosa* strains isolated in Switzerland, Denmark and Germany. Protein domain architecture revealed that *MexB* gene proteins were involved in particular efflux pump function. Protein sequences aligned by multiple sequence alignment revealed conserved regions and sequence variants, which suggested antibiotic translocation and evolutionary divergence. These highly conserved regions could be used for diagnostic purposes of efflux pump *MexB* genes.

**Conclusion:**

To avoid their spread in hospital settings, responsible authorities ought to begin rigorous initiatives in order to reduce the prevalence of multi-drug resistant, extensively drug resistant, and efflux pump carrying isolates in clinical settings.

## 1 Introduction

A common source of nosocomial infections in patients is the gram-negative bacterial pathogen *P. aeruginosa* (Reynolds and Kollef, 2021). It is estimated that microbial biofilms account for 60%–70% of hospital acquired illnesses. The pathophysiology of biofilm infection includes the immune response to biofilms, which causes collateral harm to nearby tissues (Moser et al., 2021). Dental caries, periodontitis, otitis media, chronic sinusitis, persistent wound alterations, musculoskeletal infections (osteomyelitis), biliary tract infections, bacterial prostatitis, native valve endocarditis, and infections connected to medical devices are among the infections linked to biofilms (Tuon et al., 2022). *P. aeruginosa* secretes virulent factors such as lipopolysaccharides and outer membrane proteins to adapt to harsh conditions. These factors help with host cell adhesion, tissue damage, and resistance to antibiotics (Qin et al., 2022). *P. aeruginosa* has intrinsic resistance mechanisms such as low outer membrane permeability, the presence of β-lactamases such as *OXA-50*, *AmpC*, and antibiotic efflux pumps (Langendonk et al., 2021).

Antibiotic resistance in *P. aeruginosa* has been linked to four primary sets of efflux pumps: *MexAB-OprM*, *MexXY*, *MexCD-OprJ*, and *MexEF-OprN*. One well-known and common intracellular mechanism of clinical aminoglycoside resistance in *P. aeruginosa* is the drug-inducible *MexXY*-*OprM* (Dey et al., 2020). *MexA*, *MexD*, *MexE*, and *MexY* gene overexpression has been linked to resistance to ceftazidime, imipenem, and ciprofloxacin (Abavisani et al., 2021). *MexAB-OprM*, the first multidrug efflux pump found in *P. aeruginosa*, is pointed out to be the main contributor to antibiotic resistance in the species. A rise in drug concentration near the pump causes *MexB* to change its shape, allowing it to eject active molecules toward the periplasmic tunnel and outer membrane that *MexA* and *OprM* have formed (Lorusso et al., 2022). Strong efflux pump expression *MexAB-OprM* plays a major role in *P. aeruginosa*’s resistance to carbapenems (Pan et al., 2016). A comprehensive phenotypic and genotypic approach has been developed to identify *Mex*-mediated efflux pumps, validated using reference strains, and assessed for clinical relevance (Mesaros et al., 2007). In order to help manage epidemics in crucial patient management areas, whole-genome sequencing can offer comprehensive information in a clinically appropriate time (Lauritsen et al., 2021). Antibiotic resistance genes including *MexB, MexF*, and *MexY* were found in resistant isolates of *P. aeruginosa* (Chettri et al., 2023). Higher homology between *MdtB* with *MuxB* and *MdtC* with *MuxC* is observed in the two-Resistance-nodulation-division (RND) subunit systems of *E. coli* and *P. aeruginosa* (Górecki and McEvoy, 2020). Single nucleotide polymorphisms in efflux pumps and porins have been linked in recent research to the emergence of MDR clones that pose a significant risk (Aguilar-Rodea et al., 2022). *P. aeruginosa*’s *MexB* gene study uses the Ramachandran plot to assess the structural stability and appropriate folding of the protein, which are essential for the protein’s role in antibiotic resistance (Sennhauser et al., 2009).

This study aimed to assess the burden of *P. aeruginosa* isolates in medical implants associated infections, their resistant status, abundance of *MexB* efflux pump genes, association between multidrug-resistant (MDR) and extensively drug-resistant (XDR) isolates with *MexB* genes. The nucleotide sequence of *MexB* genes was determined and analyzed evolutionary relationships, which is critical in epidemiology. The significance of *MexB* structural and functional domains in antibiotic resistance was shown by the structural and domain study of the protein in isolates. It gave important information to generate inhibitors to fight resistant *P. aeruginosa* infections and describes how manipulations in these domains can increase efflux activity, which contributes to MDR.

## 2 Materials and methods

A total of 42 samples of *P. aeruginosa* were isolated from patients with medical implant associated infections. Samples were processed in microbiology laboratory, COMSATS University Islamabad using the standard operating procedures.

### 2.1 Isolation and identification

The collected samples were grown on Blood agar (Oxoid, UK) and MacConkey agar medium (Oxoid, UK). As directed by the manufacturer, 40 g of blood agar base powder was dissolved in 1 L of distilled water. For 15 min, the solution was autoclaved at 121°C. The sterile-defibrinated blood was added to the medium until it reached a final concentration of 5% (v/v) at 45°C–50°C after it has cooled to roughly 45°C–50°C. The samples were identified by colony morphology, by gram staining, and by using biochemical tests (Bonnet et al., 2020). The reference strain was *P. aeruginosa* ATCC 27853. Matrix-assisted laser desorption ionization–time of flight (MALDI-TOF) (bioMerieux VITEK MS, France) was used for the automatic identification. Using MALDI-TOF technology, VITEK MS is an automated mass spectrometry microbial identification system that can offer single-choice identifications at the species and genus levels in a matter of minutes. The extended database accurately detects 1,316 species, with an average of 12 strains/species spanning microbiological and technological variability (VITEK MS V3.2.0 IVD CE-marked database). After preparation, bacterial colony from 24-h fresh culture, deposited with a 1 µL of calibrated loop, was inoculated on the target slide, and 1 µL of α-CHCA matrix was used, and the target slide is placed in a high-vacuum setting. The sample is ionized by a precise laser burst; an electric charge releases and accelerates a “cloud” of proteins; the proteins’ TOF is calculated using a formula from the time recorded after they have passed through the ring electrode. The results were observed and noted using Myla software (Rakotovao-Ravahatra et al., 2021; Moehario et al., 2021).

### 2.2 Antimicrobial susceptibility testing

Antimicrobial susceptibility testing was done using Kirby-Buyer disk diffusion method. The results of antimicrobial susceptibility testing were interpreted using CLSI M100Ed33E (Clinical & Laboratory Standards Institute) (CLSI 2023). The following antibiotics with respective concentrations were tested: piperacillin + tazobactam (100/10 µg), cefoperazone (30 µg), cefotaxime (30 µg), cefepime (30 µg), gentamicin (10 µg), amikacin (30 µg), imipenem (10 µg), meropenem (10 µg), doxycycline (30 µg), tigecycline (10 µg, broth dilution assay), ciprofloxacin (5 µg), levofloxacin (5 µg), polymyxin B (10 µg, broth dilution assay), and colistin (10 µg, broth dilution assay) (Oxoid, Basingstoke, UK).

### 2.3 Minimum inhibitory concentration

All tested antibiotics’ MICs were determined using the automated VITEK 2 compact system, which is in accordance with Clinical and Laboratory Standards Institute (CLSI) M100Ed33E breakpoints (Clinical and Laboratory Standards Institute, 2023). Colistin’s minimum inhibitory concentration (MIC) was obtained by the broth microdilution method using the European Committee on Antimicrobial Susceptibility Testing (EUCAST) breakpoints (Turnidge and Abbott, 2022). The antibiotics that were used in the under mentioned concentration range were cefoperazone, cefotaxime, cefepime, amikacin, ciprofloxacin, levofloxacin, gentamicin (0.5–256 µg/mL), meropenem, imipenem (0.06–32 µg/mL), piperacillin + tazobactam (0.5–512 µg/mL), tigecycline (0.125–128 μg/mL), and colistin (0.25–4 µg/mL).

### 2.4 Molecular detection of *MexB* genes and Sanger sequencing

DNA extraction was done by using Qiagen DNA Mini kit. Primers of *MexB* gene, F: 5′-GTGTTCGGCTCGCAGTACTC-3′ and R: 5′-AACCGTCGGGATTGACCTTG-3′, with annealing temperature of 56°C were used. The *MexB* genes amplified in same position as previously reported (Kishk et al., 2020). Agarose (w/vol.; 1.5%) containing ethidium bromide (0.5 mg/mL; Qiagen, Germany) was utilized for the agarose gel electrophoresis, and a 100-bp DNA ladder was employed as the size marker. Negative controls were devoid of a DNA template. Sequencing of *MexB* gene was done using Sanger sequencing. Sequence reads having *MexB* genes were subjected to BLAST analysis (NCBI), and the sequences were deposited in GenBank.

### 2.5 MIC to show presence of efflux pump

For every MDR and XDR *P. aeruginosa* isolate, the MIC of meropenem was assessed. Subsequently, 10 μg of carbonyl cyanide m-chlorophenyl hydrazone (CCCP) was added to each Mueller–Hinton agar plate that contained the antibiotics (0.5 to 128 µg/mL). In the Mueller–Hinton agar, the final concentration of CCCP was 25 µg/mL. A plate containing CCCP without any antibiotics was used as control. Any antibiotic with CCCP that reduces the MIC by two to four times suggests that the isolates have an active efflux pump (Kishk et al., 2020).

### 2.6 Phylogenetic analysis

MEGA X software (https://www.megasoftware.net) was used to do the phylogenetic analysis of the target sequences. First, the sequences were aligned using the ClustalW algorithm to ensure consistency in sequence comparison (Kumar et al., 2018). After alignment, the neighbor-joining method was used to build a phylogenetic tree. One thousand bootstrap repetitions were used to evaluate the tree’s resilience.

### 2.7 MolProbity score analysis and 3D structural modeling

By using SWISS-MODEL (https://swissmodel.expasy.org), the 3D structural models of the proteins were created (Robin et al., 2024). Using the MolProbity web server (https://molprobity.biochem.duke.edu), the precision and accuracy of the selected structures were verified by evaluating all-atom contacts and geometrical outliers (Williams et al., 2018). Ramachandran map was used, produced by MolProbity, which highlights permissible and prohibited areas for the protein’s backbone dihedral angles and structural validations.

### 2.8 Protein domain analysis using InterPro

The InterPro database (https://www.ebi.ac.uk/interpro) was used to find functional domains within the translated protein sequences. To anticipate domains, family classifications, and functional locations within protein sequences, InterPro combines a variety of protein family databases, including Pfam, PRINTS, and PROSITE. Understanding the biological function of proteins and their functioning is made easier by this approach. Protein sequences were first submitted to the InterPro database so that they could be scanned. The platform’s integrated tool InterProScan was utilized to conduct a search across all member databases in order to find conserved domains, motifs, and families connected to the protein (Richardson et al., 2019).

### 2.9 Statistical analysis

The statistical analysis was performed by IBM SPSS statistics 20, and GraphPad Prism 9.0 software. All experiments were performed in triplicate, and paired sample t-test and chi-squared test were used to analyze data. Statistical significance value was set at p ≤ 0.05.

## 3 Results

### 3.1 Isolation and identification

A total of 42 isolates of *P. aeruginosa* were isolated from medical implants infectious samples. Colony morphology, biochemical tests, and MALDI-TOF analysis confirmed the presence of *P. aeruginosa* as shown in **Supplementary Figures S1A–D**.

### 3.2 Antimicrobial susceptibility testing

The antimicrobial susceptibility profiling showed that the isolated *P. aeruginosa* was resistant to gentamycin (66%), amikacin (66%), cefoperazone (67%), cefotaxime (64%), ciprofloxacin (62%), cefipime (62%), pipracillin-tazobactum (52%), meropenem (54%), and imipenem (54%), whereas the most effective antibiotics were tigecyclines and colistin as displayed in **Table 1**. The resistant status against different antibiotics revealed that 24/42 (57%) were MDR isolates resistant to more than three classes of antibiotics including penicillins, cephalosporins, aminoglycosides, flouroquinolones; whereas 5/42 (12%) were XDR as they showed resistance against penicillins, cephalosporins, aminoglycosides, flouroquinolones, and carbapenems classes of antibiotics and showed sensitivity against only two classes: polymyxins and tetracyclins. The 13/42 (31%) isolates were sensitive to all classes of antibiotics as shown in **Supplementary Table S1**. The percentage (%) of MDR, XDR, and sensitive isolates are shown in **Table 2**.

**Table 1:** Antibiotic susceptibility profiling of *P. aeruginosa*.

Antibiotics	Number of resistant isolates (%)	Number of sensitive isolates (%)
Piperacillin-Tazobactum (PIP-TAZ)	22 (52%)	20 (48%)
Cefperazone (CPZ)	28 (67%)	14 (33%)
Cefotaxime (CTX)	27 (64%)	15 (36%)
Cefipime (FEP)	26 (62%)	16 (42%)
Meropenem (MEP)	23 (55%)	19 (45%)
Imipenem (IPM)	23 (55%)	19 (45%)
Gentamycin (GEN)	28 (66%)	14 (34%)
Amikacin (AK)	28 (66)	14 (34%)
Tigecycline (TG)	0 (0)	42 (100%)
Ciprofloxacin (CIP)	26 (62)	16 (38%)
Levofloxacin (LEV)	23 (55)	19 (45%)
Colistin (CT)	0 (0)	42 (100%)
Polymyxin B (PB)	0 (0)	42 (100%)

**Table 2:** MDR, XDR, and sensitive isolates (%).

Resistance status	Number of isolates (%)
MDR	24 (57%)
XDR	5 (12%)
Sensitive	13 (31%)
Total	42 (100%)

### 3.3 Molecular detection of *MexB* gene and Sanger sequencing

**Figure 1:**
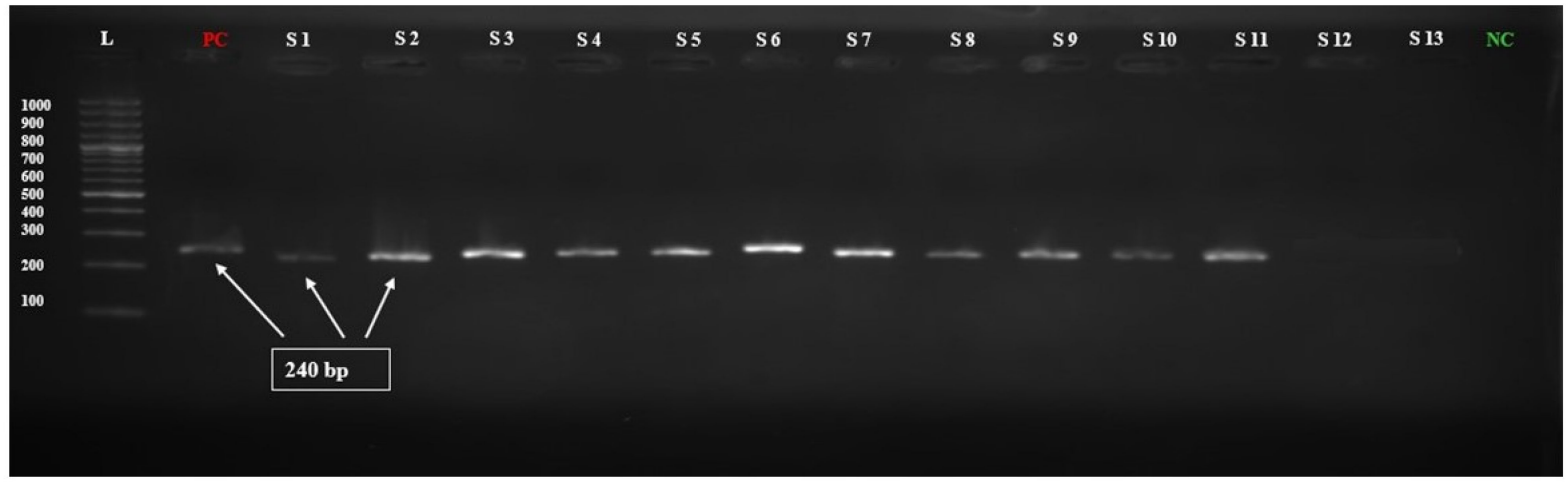
Electrophoresis gel picture of amplified Mex-B gene.

Molecular detection of *MexB* genes was done by polymerase chain reaction in 42 isolates. All resistant isolates (n = 29) revealed the presence of *MexB* genes with 244 bp, whereas *MexB* gene was absent in sensitive isolates. Furthermore, the *MexB* gene presence in positive control, selected isolates of MDR sample 1 to sample 6 (S1–S6), XDR sample 7 to sample 11 (S7–S11), and absence in two sensitive isolates (S12–S13) are shown in **Figure 1**. The selected isolates of MDR (S1–S6) and XDR (S7–S11) were further processed for Sanger sequencing, and the sequencing reads of MDR (S1–S6) and XDR (S7–S11) isolates were submitted to NCBI. The accession numbers PP964894.1, PP964895.1, PP964896.1, PP964897.1, PP964898.1, PP964899.1, PP964900.1, PP964901.2, PP964902.1, PP964903.1, and PP964904.1 of these MDR (S1–S6) and XDR (S7–S11) isolates with their isolation sources are shown in **Table 3**.

**Table 3:** GeneBank accession numbers of *MexB* gene–positive isolates of MDR (S1–S7) and XDR (S7–S11) samples.

Isolate ID	Sample sites	Accession numbers
S1	Pus from left ankle implant	PP964894
S2	Pus from knee joint	PP964895
S3	Pus from prosthetic leg implant	PP964896
S4	Pus from hip implant	PP964897
S5	Nail in femur	PP964898
S6	Screws in femur	PP964900
S7	Interlocking nails of femur	PP964901
S8	Pus from knee joint	PP964902
S9	Pus Knee joint	PP964903
S10	Pus from proximal femoral nail	PP964904
S11	Screws in tibial plateau	PP964905

### 3.4 MIC for the efflux pump detection

**Figure 2:**
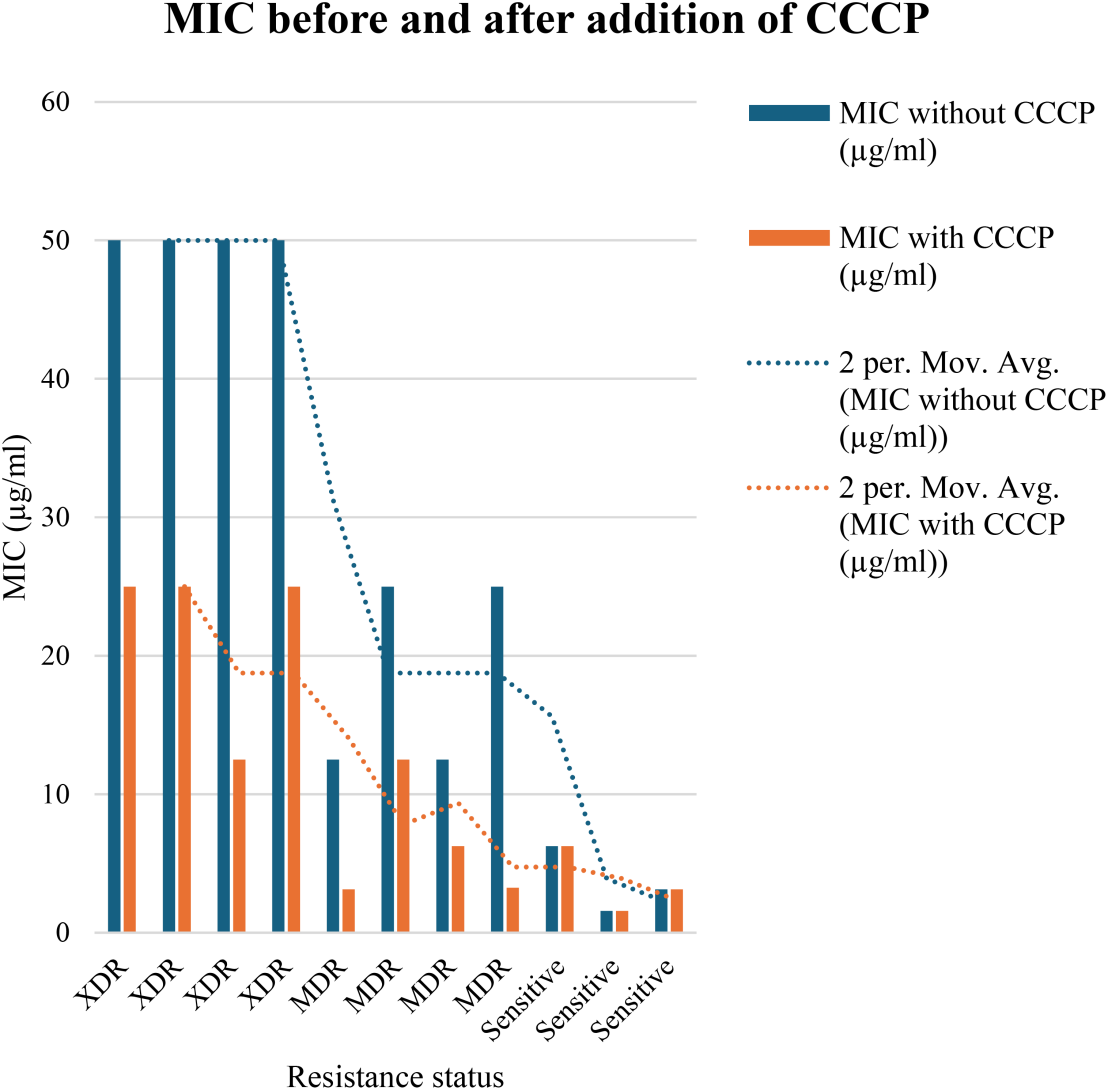
In 4 MDR and 4 XDR isolates of *P. aeruginosa*, the MIC was significantly decreased after addition of CCCP, In case of 4 MDR isolates, 2-fold, 3-fold and 4-fold reduction was observed, while in case of 4 XDR isolates 2-fold and 3-fold reduction was observed after addition of CCCP (p<0.05). No MIC changes were observed in 3 sensitive isolates (p>0.05) indicated that active efflux pump was not present in these isolates.

**Figure 3:**
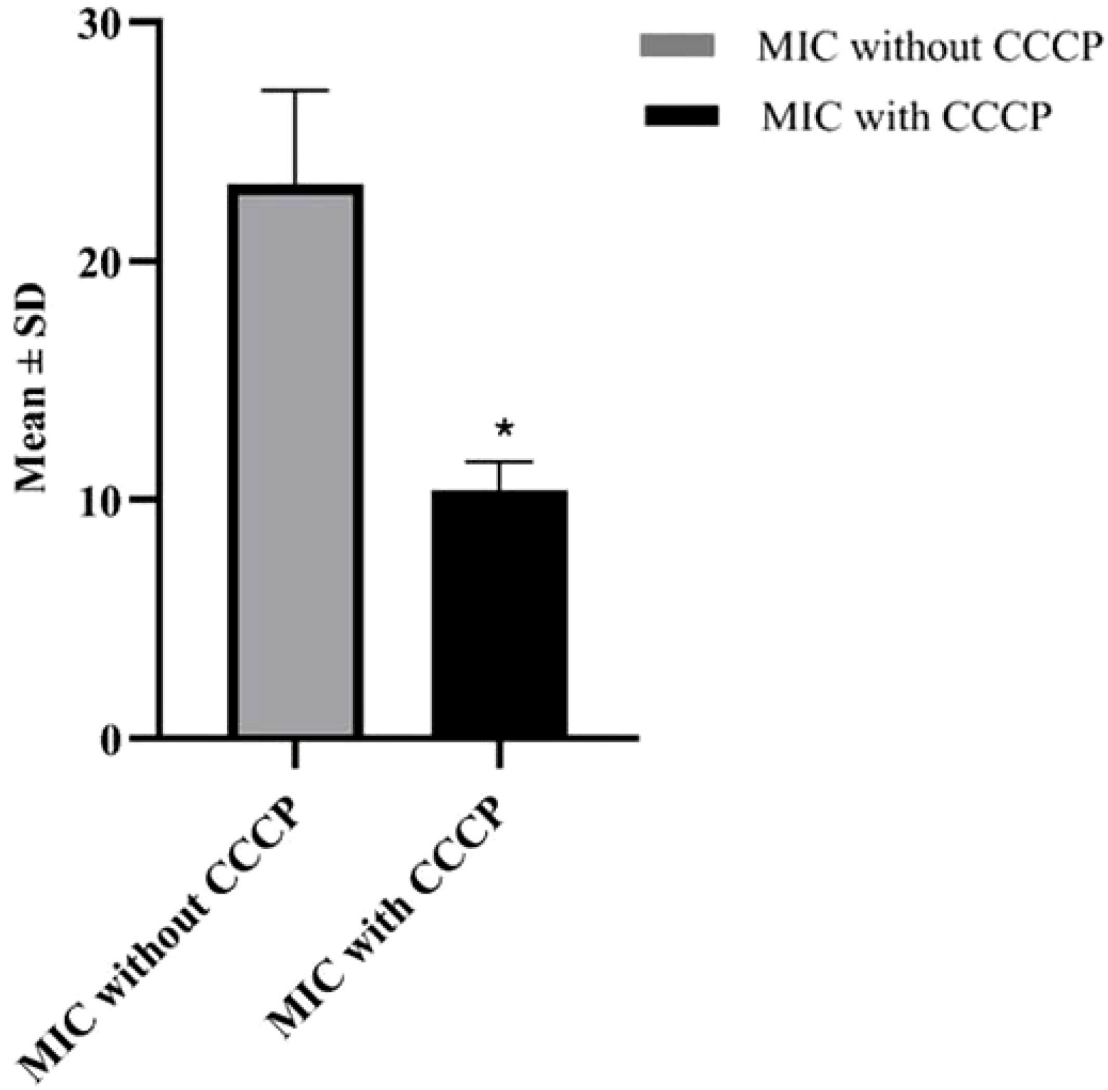
Mean and Std. error difference of MIC with CCCP (p<0.05) and without CCCP. * showing significant difference.

In order to evaluate the presence of active efflux pump, MIC in the presence of CCCP and meropenem (antibiotic) was investigated. A significant reduction (p < 0.05) in MIC was observed in meropenem-resistant isolates, and no MIC changes were observed in sensitive isolates; paired sample t-test was performed to evaluate the MIC differences, as shown in **Figure 2**. The mean difference was 14.761, and the Std. error difference was 6.803 calculated by independent sample t-test as shown in **Figure 3**.

### 3.5 Phylogenetic analysis

**Figure 4:**
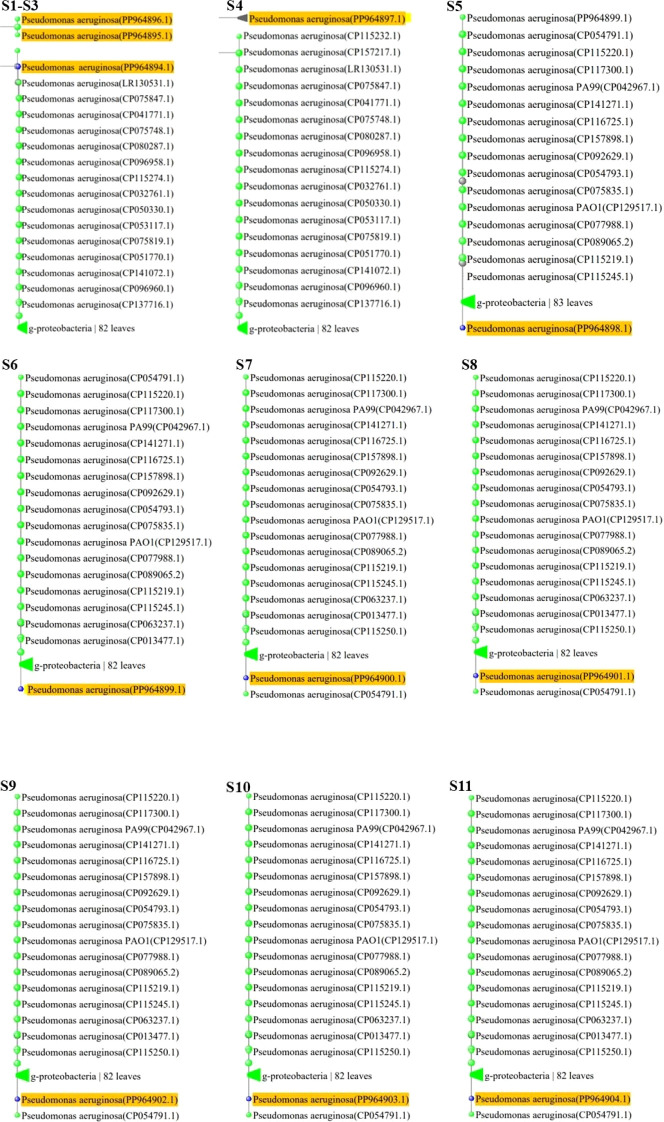
(S1-S11) Phylogenetic analysis of MDR and XDR *P. aeruginosa* isolates. Yellow color highlights accession numbers of submitted Mex-B genes in Gene bank. Labels like “g-proteobacteria “82 leaves” and “83 leaves” indicates the number of taxa or sequences included in each group, showing diversity within these classifications.

The BLAST MEGA X software (https://www.megasoftware.net) and ClustalW algorithm alignment tool were used to locate similar nucleotides between the sequences of the efflux transporter gene *MexB*, from *P. aeruginosa*, with simultaneous comparisons to other strains and recorded genes. The evolutionary connections between the 11 isolated *P. aeruginosa* strains in this investigation and the 19 reference strains are displayed in this phylogenetic tree. Phylogenetic analysis showed the closest similarities of the samples S1–S3 with LR130531.1 (submitted 12 November 2018, Biozentrum, University of Basel, Switzerland), S4 with CP115235.1 (submitted 27 December 2022, Technical University of Denmark), S5 with CP115245.1 (submitted 27 December 2022, Technical University of Denmark), S6 with CP013477.1 (submitted 7 December 2015 Bioinformatics, Leibniz Institute DSMZ, Inhoffenstr. Germany), S7 with CP115250.1 (submitted 27 December 2022, Technical University of Denmark), and S8–S11 with CP115250.1 (submitted 27 December 2022, Technical University of Denmark). The number of sequences (referred to as “leaves”) slightly varies across datasets. In each tree, the highlighted *P. aeruginosa* strains are shown as part of a larger phylogenetic context within g-proteobacteria. The differences across these phylogenetic trees (S1–S11) likely represent variations in genetic makeup, evolutionary histories, and environmental influences on the *P. aeruginosa* strains. The divergence in highlighted strains reflects evolutionary pressures, such as antibiotic resistance and adaptation to specific environments. Close clustering of highlighted strains (S1–S3) shared evolutionary history while highlighted strains (S5–S11) are farther apart, suggesting divergent lineages as shown in **Figure 4** (S1–S11).

### 3.6 Clustal Omega

**Figure 5:**
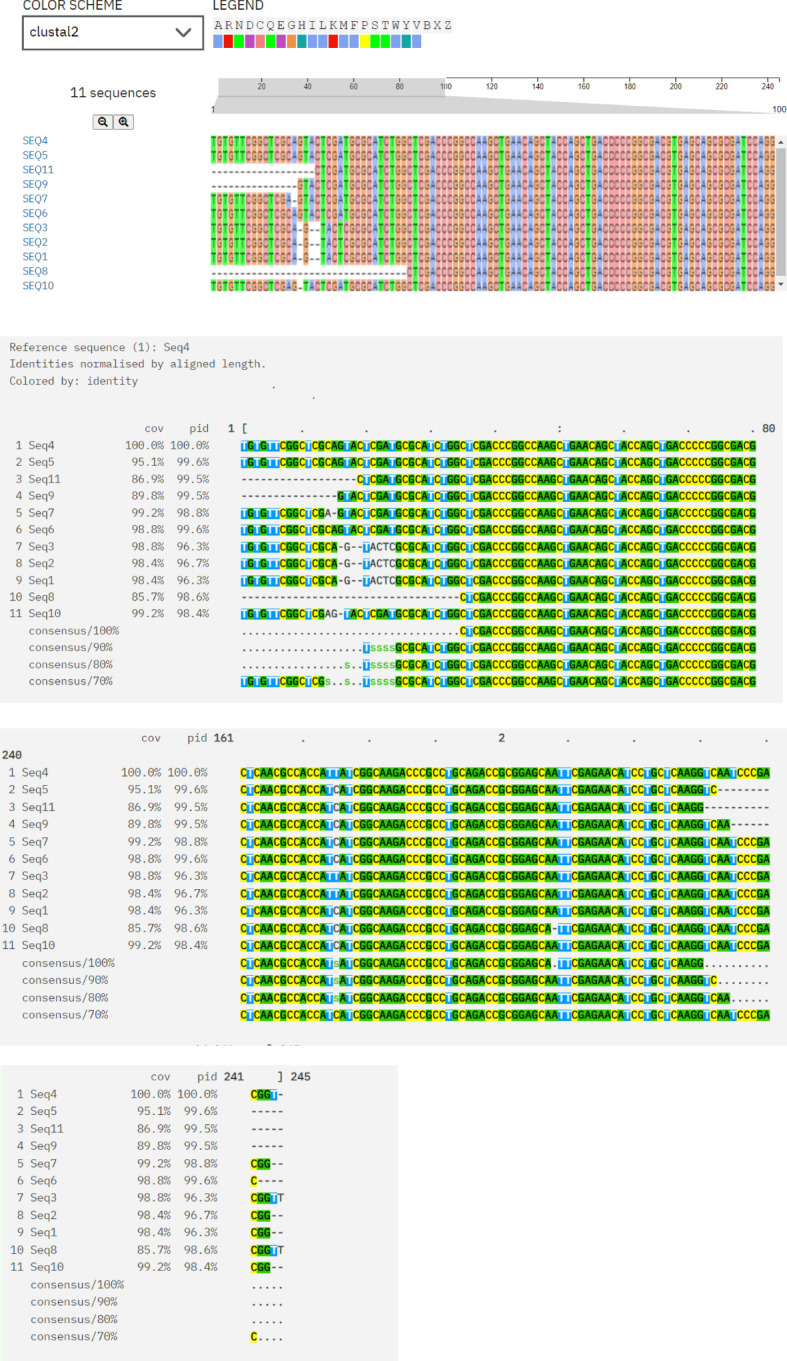
Multiple sequence alignment of protein sequences using ClustalW Omega. “A and T” within blue color in all sequences showing hydrophobic domains. “C and G” within Magenta color boxes showing negatively charged amino acids. “T, G, A” within green color showing polar amino acids. “C” within pink color showing cystine amino acids. “C” in yellow colors showing prolines. “A” in cyan color showing aromatic amino acids. White and colorless area showing nonconserved regions having variability. In every sequence, conserved areas are continuously highlighted in different colors (green, yellow and pink) representing designated amino acids.

Multiple sequence alignment of protein sequences was done using Clustal Omega, which indicates conserved regions and sequence variations. Conserved areas shown as sequences exhibited total conservation at regions where identical residues (such as nucleotides and amino acids) match. In every sequence, these points are continuously highlighted in different colors (green, yellow, and pink), representing designated amino acids. Sequence 4 represents the baseline sequence for comparison, as evidenced by its 100% identity and coverage. Conserved regions that could be involved in substrate binding and the antibiotics’ translocation from the bacterial cell and sequence variations are indicative of evolutionary divergence, adaptation to different environments, and exposure to various antibiotics. Variability is seen in gaps, substitutions, and mismatches between the sequences as area contains gaps (dashes: -) or replacements in some sequences (e.g., Seq3, Seq6, and Seq8). Variability might be a sign of functional specialization, species-specific adaptations, or evolutionary divergence. The majority of sequences show limited variability between areas and fit well with the reference (≥90% coverage/identity) as shown in **Figure 5**.

### 3.7 MolProbity score analysis and 3D structural models of protein sequences, alongside their respective Ramachandran plots

The entire quality of the structure, considered both geometry and steric, is gauged by the MolProbity score. A greater quality is indicated by lower scores. High-quality models are suggested by MDR samples S4–S6 and XDR samples S7–S11 with a MolProbity score of 0.50. MDR samples S1–S3 had a higher score of 1.42, although still within acceptable range bounds. There were no steric collisions in any of the samples, with a clash score of 0.00. Favored regions were shown by Ramachandran plot analysis. More than 98% of residues in favored regions correspond to high-quality models, ideally. All residues were found in the preferred locations in XDR samples S8–S11. Results for MDR samples S4–S6 and XDR samples S7–S11 were likewise excellent, with most of them exceeding 98%. Although the favored values of MDR samples S1–S3 were slightly lower, i.e., 95.59, 93.59%, and 97.59%, respectively, they were still within acceptable ranges. The findings of Ramachandran outliers revealed excellent structural quality as indicated by the 0.00% outliers in all samples. Rotamer deviations measured variations in side-chain conformations from the predicted ones and the rotamer outliers in MDR samples S1–S4 and XDR sample S10 were 0.00%, indicating extremely high side-chain accuracy. Slightly higher values of 4.55% and 5.00%, respectively, were seen in MDR samples S1–S3 and XDR sample S11. C-Beta deviation backbone distortions indicated that majority of samples exhibited zero or slight variation, indicating good accuracy of protein structure and excellent quality of protein models. Significant deviations from the optimal bond lengths and angles were counted in the bad bonds and bad Angles section. Each sample had one to four bad angles identified, which is a small and acceptable number of improper angles, which assessed the good geometry of a protein structure and favorable steric interactions. There were no large bad bonds observed. All MDR samples S1–S6 and XDR samples S7–S11, showing Cis prolines, indicated excellent signaling, structural integrity, and interactions with other molecules. All the analyzed values of MolProbity score, clash score, Ramachandran favored, Ramachandran outliers, C-Beta deviations, bad angles, and Cis prolines are mentioned in **Table 4**.

**Table 4:** Analysis of MolProbity score, clash score, Ramachandran favored, Ramachandran outliers, C-Beta deviations, bad angles, and Cis prolines in MDR (S1–S6) and XDR (S7–S11) isolates.

Sample number	MolProbity score	Clash score	Ramachandran favored	Ramachandran outliers	Rotamer outliers	C-Beta deviations	Bad bonds	Bad angles	Cis prolines
**S1**	1.42	0.00	95.59%	0.00%	4.55%	1	0/615	2/835	1/4
**S2**	1.42	0.00	93.59%	0.00%	4.55%	1	0/615	2/835	1/4
**S3**	1.42	0.00	97.59%	0.00%	4.55%	1	0/615	2/835	1/4
**S4**	0.50	0.00	98.73%	0.00%	0.00%	0	0/623	4/845	1/4
**S5**	0.50	0.00	98.67%	0.00%	0.00%	0	0/595	1/806	1/3
**S6**	0.50	0.00	98.72%	0.00%	0.00%	0	0/619	3/840	1/4
**S7**	0.50	0.00	98.65%	0.00%	0.00%	0	0/585	3/794	1/4
**S8**	0.50	0.00	100.00%	0.00%	0.00%	0	0/417	2/566	1/3
**S9**	0.50	0.00	98.57%	0.00%	0.00%	0	0/557	1/755	1/3
**S10**	0.50	0.00	98.65%	0.00%	0.00%	0	0/585	3/794	1/4
**S11**	0.50	0.00	100.00%	0.00%	5.00%	1	0/537	4/727	1/3

**Figure 6:**
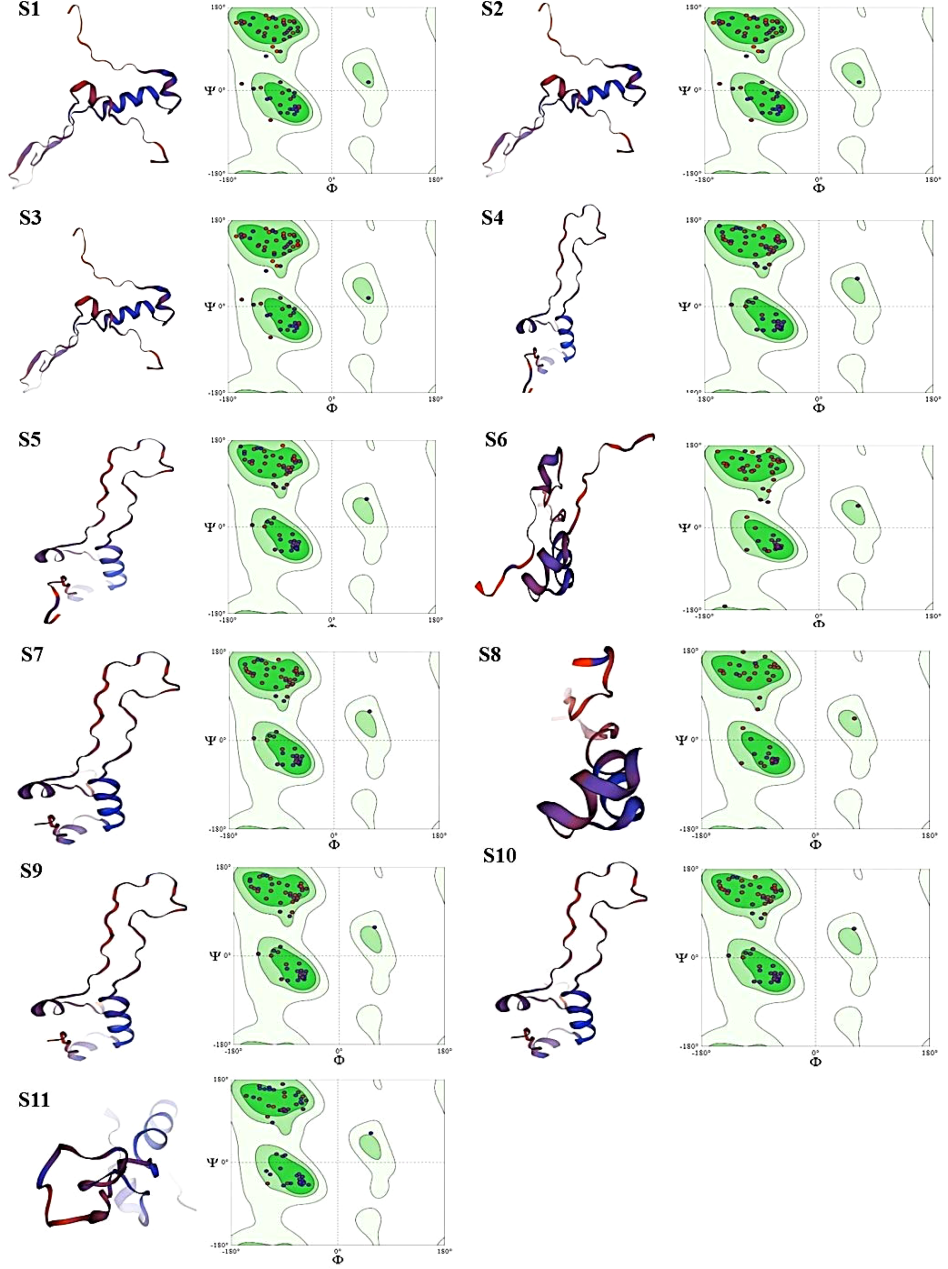
Images (S1-S11) display the 3D structural models of protein sequences, alongside their respective Ramachandran plots. Each panel has two main parts: Left Part: containing ribbon representation of a protein structure. Red/Orange: loops showing unstructured regions (random coils). Blue/Purple: represents Alpha-helices and beta sheets. Right Part: containing Ramachandran plot. Green Regions (in Ramachandran plots): showing allowed regions for specific phi (Φ) and psi (Ψ) dihedral angles, indicating favorable conformations for protein backbones. Dots in Ramachandran Plots: represent actual phi/psi angles of residues within the proteins so, according to our findings MDR samples S1-S3 showing sterically unfavorable angles are indicated by a considerable number of residues that fall outside of the permitted areas in Ramachandran plot, implying inaccurate conformations and model errors. MDR samples S4-S6 and XDR samples S7-S11 showing sterically favorable angles are indicated by a considerable number of residues that fall within the permitted areas and exhibited excellent 3D structures and showed the proteins adopted a conformation that reflects its biological function, accurately reproducing secondary (α-helices, β-sheets) and tertiary structures.

According to the analysis, MDR samples S4–S6 and XDR sample S11 were found to be of the highest quality, whereas XDR sample S8 showed perfect Ramachandran favored values and no outliers. MDR samples S1–S3 were found to be an average quality, with slightly higher MolProbity scores and lower favored Ramachandran values; these samples also showed unfavorable angles, indicated by a considerable number of residues that fall outside of the permitted areas, implying inaccurate conformations or model errors. MDR samples S4–S6 and XDR samples S7–S11 exhibited excellent 3D structures and showed the proteins adopted a conformation that reflects its biological function, accurately reproducing secondary (α-helices and β-sheets) and tertiary structures as shown in **Figure 6**.

### 3.8 Protein domain analysis

The output illustrates the identification of functional domains within the translated protein sequences. The translated protein sequences were analyzed by InterPro, as shown in **Supplementary Figure S2** (MDR S1–S6 and XDR S7–S11), suggesting that the analyzed protein sequence belongs to the Acriflavin resistance protein family (AcrR) and is associated with the AcrB transporter family, which is part of a well-known multidrug efflux system. The presence of the AcrB DN/DC subdomain within the homologous superfamily indicates the role of this protein in docking and interacting with the outer membrane channel TolC, forming a critical part of the efflux pump system. The identified domains and families, particularly the AcrB transporter and its docking domain, are key players in antibiotic resistance in *P. aeruginosa* isolates. This efflux system is a significant contributor to its resistance profile, helping it to survive in the presence of various antibiotics by actively expelling them.

## 4 Discussion

In the present study, the *P. aeruginosa* isolates from medical implants exhibited highest resistance against gentamycin, amikacin, and cefepime but lowest resistance against meropenem and imipenem. The most useful drug was polymyxin B followed by tigecycline. A study from 18 European countries, regarding antimicrobial resistance, showed that *P. aeruginosa* strains exhibited the highest degrees of resistance against ampicillin, gentamycin, ciprofloxacin, and amikacin, whereas imipenem, meropenem, colistin, and tigecycline were the most effective medications (Oliver et al., 2024). According to the World Health Organization (WHO), *P. aeruginosa* is a leading cause of antibiotic resistance and was listed as a “priority pathogen” by the WHO (Balakrishnan, 2022). Because these pathogens have shown resistance to certain antimicrobials, such as imipenem, meropenem, and third-generation cephalosporins—currently, the most effective medications for fighting MDR bacteria, they have presented a significant therapeutic challenge (Mulani et al., 2019). With similar findings in New Zealand and USA, the *P. aeruginosa* is found mostly in healthcare environments and plays a crucial part in causing nosocomial infections and resistant to diverse range of antibiotics (Moradali et al., 2017) (Reynolds and Kollef, 2021). Another study in Ethiopia concluded that *P. aeruginosa* pooled prevalence of antimicrobial resistance varies depending on the antibiotic: 20.9% for amikacin, 28.64% for meropenem, and 98.72% for ceftriaxone (Asmare et al., 2024). Likewise, a study conducted in Australia and concluded that *P. aeruginosa* resistant to a wide range of antibiotic classes, such as aminoglycosides (amikacin, gentamicin, and tobramycin), fluoroquinolones (FQs; ciprofloxacin, ofloxacin, and norfloxacin), carbapenems, and tetracyclines, its potential to develop MDR is a compelling aspect of concern (Avakh et al., 2023). Above recent studies were related to clinical isolates from hospital settings, supporting our study analysis that an emerging antimicrobial resistance in *P. aeruginosa* is a critical concern in healthcare settings.

In current study, the antimicrobial susceptibility profiling of *P. aeruginosa* revealed that 57% of isolates were MDR, 12% were XDR and 31% were sensitive. Whereas another study in Ethiopia revealed that the level of MDR was 45.9% and the XDR rate was 9.5% (Asamenew et al., 2023), this study was different in context as our study sampling source was infectious medical implants. Another study showed that 83% of *P. aeruginosa* isolates were MDR isolated from clinical samples (Mekonnen et al., 2021). Similar study results in Nigeria found that 12.8% of the isolates were MDR bacteria, and the majority of MDR strains that had high multiple antibiotic resistance indexes showed resistance to a variety of antibiotics, such as aminoglycosides, third- and fourth-generation cephalosporins, β-lactams, and FQs, which is constantly increasing (Adejobi et al., 2021). According to the INFORM database, a study in Spain found that *P. aeruginosa* that is XDR and MDR is a common and difficult nosocomial pathogen with consistently high rates that range from 9.0% to 11.2% and 11.5% to 24.7%, respectively (Recio et al., 2020).

The *MexB* gene, which encodes part of the *MexAB-OprM* efflux pump in *Pseudomonas aeruginosa*, is often considered more significant than its counterparts *Mex-A* and *OprM* because of its direct role in drug transport and efflux, contributing to antimicrobial resistance. That is why we only focused on *Mex-B* genes. According to the results of the study by Piddock (2006) and Tsutsumi et al. (2019), *MexB* is the active transporter in this tripartite complex, playing the most crucial role in the efflux of antibiotics. It directly binds and extrudes drugs from the bacterial cytoplasm and periplasm to the outside environment. *MexA* and *OprM* are structural components that aid the pump’s function, but, without *MexB*, the efflux pump cannot transport drugs, making *MexB* the critical efflux driver. In our study, *MexB* gene was identified in 69% resistant isolates and also found that it was a major factor in antibiotic resistance, with similar findings by studies in China, Switzerland, and France that *AB-OM porin M (MexAB-OprM)* and *MexXY-OprM* are multidrug efflux proteins that have been thoroughly investigated for their significant contributions to MDR. Moreover, these efflux pumps have unique resistance mechanisms that could result in the formation of XDR or PDR phenotypes and highly resistant strains (Qin et al., 2022; Dreier and Ruggerone, 2015; Compagne et al., 2023). Another research finding in Nepal concluded that 94.4% of *P. aeruginosa* isolates were harboring *MexB* genes, but their association with antimicrobial resistance was not analyzed (Sharma et al., 2023).

In current study, we have confirmed the presence of *MexB* gene efflux pump by CCCP assay; likewise, the previous study by Ahmed et al. (2021) examined the efflux pump expression under the heading of “Determination of Efflux Pumps Expression in Resistant Isolates”: The MICs of ciprofloxacin, meropenem, amikacin, and ceftriaxone were detected for 25 MDR *P. aeruginosa* isolates by agar dilution method in the presence and absence of efflux pump inhibitor CCCP (Sigma, San Jose, CA, USA) at a final concentration of 10 μM. A four-fold reduction in MIC or more of the tested antibiotics after adding CCCP is an indication for the presence of efflux pumps. The role of efflux pump *MexB* genes in antimicrobial resistance was confirmed by adding CCCP with antibiotics, because there was a significant reduction observed in MIC following the addition of CCCP reagents with antibiotics. Likewise, the results in France on multiple gram-negative organisms show that MIC was evaluated with and without CCCP and a two-fold decrease of colistin MIC was calculated for each strain. There was a significant decrease observed in MIC after addition of CCCP (Baron and Rolain, 2018). Recently, similar results were examined in Iran: the expression of the *Mex* efflux pump in 122 clinical isolates of *P. aeruginosa*. This study found a substantial association between MexB expression and resistance to antipseudomonal drugs, with *MexB* (69%) expression emerging as the second most active efflux pump (Rahbar et al., 2021). A research study in China revealed a significant decrease in MIC, following the CCCP addition against colistin-resistant bacteria (Ding et al., 2023). The study results of the abovementioned studies showed similarities with our results in the context that MDR-to-XDR ratio in clinical isolates is increasing and that *MexB* had a strong relationship with antimicrobial resistance, but there is a limitation that no one analyzes the molecular and structural analysis of their clinical isolates.

In this study, partial sequenced data were submitted to GenBank, and accession number was assigned against each submitted MDR and XDR isolates of *P. aeruginosa.* The phylogenetic analysis was performed to find the similarities with isolates having a strong efflux pump system, which showed that 100% similarities were found with strains isolated from Switzerland, Denmark, and Germany, whereas a research study done in India performed phylogenetic tree analysis of *MexB* genes and also found similarities with some Asian isolates, but they did not process these samples for further structural analysis (Chettri et al., 2023). With similar study results in Japan and Belgium, the phylogenetic analysis showed that similar isolates having RND multidrug efflux pumps exhibited critical role in the resistance of gram-negative organisms. The RND efflux pumps are the most important of the several efflux pump families found in *P. aeruginosa* that are connected to MDR. RND pumps’ distinct structure and function make them essential for protecting against antibiotics (Yamasaki et al., 2023; Avrain et al., 2013).

In our study, the Ramachandran plot showed values in favored regions. More than 98% of residues in favored regions correspond to high-quality models, ideally. The findings of Ramachandran outliers revealed excellent structural quality, as indicated by the 0% outliers in all analyzed samples. In another study done in Malaysia, the subunit of efflux pump, *Amr-B* protein, indicated that the predicted model was of high quality and had appropriate backbone geometry. Amino acid T was found in the favored residue region of the Ramachandran plot, which corresponds to no steric clashes between the side chain atoms and main chain atoms (Hussin et al., 2024). Another similar study results in India on Ramachandran plot analysis program for 3D structural analysis of isolated protein models used and analyzed that 97.5% amino acids fall in the favored region and 2.5% in the allowed region, and none in the outlier region suggested an excellent model (Swain and Padhy, 2016). These study results supported our analysis scheme in a way that, for structural analysis, Ramachandran plot was the best tool to identify and interpret the best protein models.

In our study, multiple sequence alignment of protein sequences using Clustal Omega indicated conserved regions and sequence variations in partial sequence *MexB* genes. In the previous study, Clustal Omega was used by Memili et al. (2022), the resistant strain sequences were aligned with Clustal Omega Multiple Sequence Alignment program, and percent identity matrices were derived. Phylogenetic analyses to elucidate evolutionary relationships between sequences were conducted with the neighbor–end joining method through the Molecular Evolutionary Genetics Analysis (MEGAX) software, developed by the Institute of Molecular Evolutionary Genetics at Pennsylvania State University. Subsequent network analyses of the resistance gene, *vanB*, within *E. faecium* were derived from ScanProsite and InterPro. Conserved regions that could be involved in substrate binding and the antibiotics’ translocation from the bacterial cell and sequence variations are indicative of evolutionary divergence, adaptation to different environments, and exposure to various antibiotics (Sanz-García et al., 2018). A study done in Spain evaluated conserved regions and sequence variations in *P. aeruginosa* isolates but did not evaluated the MolProbity score and Ramachandran plot analysis to give insight about structural models of isolated samples (Mosquera-Rendón et al., 2016). In our study, the translated protein sequences were analyzed by InterPro, which verified that they belonged to homologous and well-established multidrug efflux transporters families, with similar findings by the study results in Iran that concluded that protein domain analysis of efflux pump genes in gram-negative bacteria showed a relationship to homologous RND families and highly conserved domains of RND MDR gram-negative bacteria (Abadi et al., 2018), yet, again, the isolation sources of samples were other than medical implants. In abovementioned studies, researchers did not highlight the details of 3D protein models of clinical isolates for target drug delivery.

## 5 Conclusion

The current study provides comprehensive information and significant understanding on occurrence MDR and XDR patterns of *P. aeruginosa* and role of *MexB* genes in resistance profiling. Phylogenetic trees of the *MexB* gene of resistant bacterial species showed clusters built on the basis of common ancestry. 3D structural modeling of protein sequences, Ramachandran plot analysis, and protein domain analysis showed excellent protein models of *MexB* genes that could be useful as potential therapeutic targets in the future medicines to predict drug design models. Sequence variations were indicative of evolutionary divergence, adaptation to different environments, and exposure to various antibiotics. Highly conserved regions were found in the multiple sequence alignment analysis that highly conserved regions could be used for the diagnostic purposes, and design primers for these domains to identify *MexB* genes in *P. aeruginosa* and eliminating or manipulating these highly conserved regions by different genetic-based methods can deactivate multidrug efflux pumps in *P. aeruginosa*.

## Data Availability

The datasets presented in this study can be found in online repositories. The names of the repository/repositories and accession number(s) can be found in the article/**Supplementary Material**.

## References

[B1] Abadi M. S. S. Gholipour A. Hadi N. (2018). The highly conserved domain of RND multidrug efflux pumps in pathogenic Gram-negative bacteria. Cell. Mol. Biol. 64, 79–83. doi: 10.14715/cmb/2018.64.13.15 30403600

[B2] Abavisani M. Goudarzi M. Ghalavand Z. Hajikhani B. Rad Z. R. Rad Z. R.. (2021). Evaluation of efflux pumps overexpression and β-lactamase genes among colistin resistant Pseudomonas aeruginosa. Gene Rep. 24, 101301. doi: 10.1016/j.genrep.2021.101301

[B3] Adejobi A. Ojo O. Alaka O. Odetoyin B. Onipede A. (2021). Antibiotic resistance pattern of Pseudomonas spp. from patients in a tertiary hospital in South-West Nigeria. Germs 11, 238. doi: 10.18683/germs.2021.1260 34422695 PMC8373413

[B4] Aguilar-Rodea P. Zúñiga G. Cerritos R. Rodríguez-Espino B. A. Gomez-Ramirez U. Nolasco-Romero C. G.. (2022). Nucleotide substitutions in the mexR, nalC and nalD regulator genes of the *MexAB-OprM* efflux pump are maintained in P. aeruginosag enetic lineages. PloS One 17, e0266742. doi: 10.1371/journal.pone.0266742 35536836 PMC9089866

[B5] Ahmed F. Y. Aly U. F. Abd El-Baky R. M. Waly N. G. (2021). Effect of titanium dioxide nanoparticles on the expression of efflux pump and quorum-sensing genes in MDR Pseudomonas aeruginosa isolates. Antibiotics 10, 625. doi: 10.3390/antibiotics10060625 34073802 PMC8225175

[B6] Asamenew T. Worku S. Motbainor H. Mekonnen D. Deribe A. (2023). Antimicrobial resistance profile of *Pseudomonas aeruginosa* from different clinical samples in Debre Tabor comprehensive specialized hospital, Northwest Ethiopia. Ethiopian J. Health Sci. 33. doi: 10.4314/ejhs.v33i3.5 PMC10416326 37576170

[B7] Asmare Z. Reta M. A. Gashaw Y. Getachew E. Sisay A. Gashaw M.. (2024). Antimicrobial resistance profile of *Pseudomonas aeruginosa* clinical isolates from healthcare-associated infections in Ethiopia: A systematic review and meta-analysis. PloS One 19, e0308946. doi: 10.1371/journal.pone.0308946 39137234 PMC11321567

[B8] Avakh A. Grant G. D. Cheesman M. J. Kalkundri T. Hall S. (2023). The art of war with Pseudomonas aeruginosa: targeting Mex efflux pumps directly to strategically enhance antipseudomonal drug efficacy. Antibiotics 12, 1304. doi: 10.3390/antibiotics12081304 37627724 PMC10451789

[B9] Avrain L. Mertens P. Van Bambeke F. (2013). RND efflux pumps in P. aeruginosa: an underestimated resistance mechanism. Antibiot. Susceptibility 26321, 26–28.

[B10] Balakrishnan V. S. (2022). WHO’s first global infection prevention and control report. Lancet Infect. Dis. 22, 1122. doi: 10.1016/S1473-3099(22)00459-5 35870460 PMC9299724

[B11] Baron S. A. Rolain J. M. (2018). Efflux pump inhibitor CCCP to rescue colistin susceptibility in mcr-1 plasmid-mediated colistin-resistant strains and Gram-negative bacteria. J. Antimicrobial Chemotherapy 73, 1862–1871. doi: 10.1093/jac/dky134 29718423

[B12] Bonnet M. Lagier J. C. Raoult D. Khelaifia S. (2020). Bacterial culture through selective and non-selective conditions: the evolution of culture media in clinical microbiology. New Microbes New infections 34, 100622. doi: 10.1016/j.nmni.2019.100622 31956419 PMC6961714

[B13] Chettri U. Nongkhlaw M. Joshi S. R. (2023). Molecular evidence for occurrence of heavy metal and antibiotic resistance genes among predominant metal tolerant Pseudomonas sp. and Serratia sp. prevalent in the Teesta River. Curr. Microbiol. 80, 226. doi: 10.1007/s00284-023-03334-9 37227565

[B14] Clinical and Laboratory Standards Institute (2023). Performance standards for antimicrobial susceptibility testing.

[B15] Compagne N. Vieira Da Cruz A. Müller R. T. Hartkoorn R. C. Flipo M. Pos K. M. (2023). Update on the discovery of efflux pump inhibitors against critical priority Gram-negative bacteria. Antibiotics 12, 180. doi: 10.3390/antibiotics12010180 36671381 PMC9854755

[B16] Dey D. Kavanaugh L. G. Conn G. L. (2020). Antibiotic substrate selectivity of P. aeruginosa and MexB efflux systems is determined by a Goldilocks affinity. Antimicrobial Agents Chemotherapy 64, 10–1128. doi: 10.1128/AAC.00496-20 PMC7526836 32457110

[B17] Ding Y. Hao J. Xiao W. Ye C. Xiao X. Jian C.. (2023). Role of efflux pumps, their inhibitors, and regulators in colistin resistance. Front. Microbiol. 14, 1207441. doi: 10.3389/fmicb.2023.1207441 37601369 PMC10436536

[B18] Dreier J. Ruggerone P. (2015). Interaction of antibacterial compounds with RND efflux pumps in Pseudomonas aeruginosa. Front. Microbiol. 6, 660. doi: 10.3389/fmicb.2015.00660 26217310 PMC4495556

[B19] Górecki K. McEvoy M. M. (2020). Phylogenetic analysis reveals an ancient gene duplication as the origin of the MdtABC efflux pump. PloS One 15, e0228877. doi: 10.1371/journal.pone.0228877 32050009 PMC7015380

[B20] Hussin A. Nathan S. Shahidan M. A. Nor Rahim M. Y. Zainun M. Y. Khairuddin N. A. N.. (2024). Identification and mechanism determination of the efflux pump subunit amrB gene mutations linked to gentamicin susceptibility in clinical Burkholderia pseudomallei from Malaysian Borneo. Mol. Genet. Genomics 299, 12. doi: 10.1007/s00438-024-02105-w 38381232

[B21] Kishk R. M. Abdalla M. O. Hashish A. A. Nemr N. A. El Nahhas N. Alkahtani S.. (2020). Efflux *MexA*B-mediated resistance in P. aeruginosa isolated from patients with healthcare associated infections. Pathogens 9, 471. doi: 10.3390/pathogens9060471 32549303 PMC7350317

[B22] Kumar S. Stecher G. Li M. Knyaz C. Tamura K. (2018). MEGA X: molecular evolutionary genetics analysis across computing platforms. Mol. Biol. Evol. 35, 1547–1549. doi: 10.1093/molbev/msy096 29722887 PMC5967553

[B23] Langendonk R. F. Neill D. R. Fothergill J. L. (2021). The building blocks of antimicrobial resistance in Pseudomonas aeruginosa: implications for current resistance-breaking therapies. Front. Cell. Infection Microbiol. 11, 665759. doi: 10.3389/fcimb.2021.665759 PMC8085337 33937104

[B24] Lauritsen J. G. Hansen M. L. Bech P. K. Jelsbak L. Gram L. Strube M. L. (2021). Identification and differentiation of Pseudomonas species in field samples using a rpoD amplicon sequencing methodology. Msystems 6, 10–1128. doi: 10.1128/msystems.00704-21 PMC8407407 34342531

[B25] Lorusso A. B. Carrara J. A. Barroso C. D. N. Tuon F. F. Faoro H. (2022). Role of efflux pumps on antimicrobial resistance in Pseudomonas aeruginosa. Int. J. Mol. Sci. 23, 15779. doi: 10.3390/ijms232415779 36555423 PMC9779380

[B26] Mekonnen H. Seid A. Molla Fenta G. Gebrecherkos T. (2021). Antimicrobial resistance profiles and associated factors of Acinetobacter and *Pseudomonas aeruginosa* nosocomial infection among patients admitted at Dessie comprehensive specialized Hospital, North-East Ethiopia. A cross-sectional study. PloS One 16, e0257272. doi: 10.1371/journal.pone.0257272 34780494 PMC8592406

[B27] Memili A. Kutchy N. Braimah O. A. Morenikeji O. B. (2022). Evolutionary conservation of motifs within vanA and vanB of vancomycin-resistant enterococci. Veterinary World 15, 2407. doi: 10.14202/vetworld. 36425127 PMC9682389

[B28] Mesaros N. Glupczynski Y. Avrain L. Caceres N. E. Tulkens P. M. Van Bambeke F. (2007). A combined phenotypic and genotypic method for the detection of Mex efflux pumps in Pseudomonas aeruginosa. J. antimicrobial chemotherapy 59, 378–386. doi: 10.1093/jac/dkl504 17289770

[B29] Moehario L. H. Tjoa E. Putranata H. Joon S. Edbert D. Robertus T. (2021). Performance of TDR-300B and VITEK® 2 for the identification of *Pseudomonas aeruginosa* in comparison with VITEK®-MS. J. Int. Med. Res. 49, 0300060521989893. doi: 10.1177/0300060521989893 33626939 PMC7925945

[B30] Moradali M. F. Ghods S. Rehm B. H. (2017). Pseudomonas aeruginosa lifestyle: a paradigm for adaptation, survival, and persistence. Front. Cell. infection Microbiol. 7, 39. doi: 10.3389/fcimb.2017.00039 PMC5310132 28261568

[B31] Moser C. Jensen P.Ø. Thomsen K. Kolpen M. Rybtke M. Lauland A. S.. (2021). Immune responses to P. aeruginosa biofilm infections. Front. Immunol. 12, 625597. doi: 10.3389/fimmu.2021.625597 33692800 PMC7937708

[B32] Mosquera-Rendón J. Rada-Bravo A. M. Cárdenas-Brito S. Corredor M. Restrepo-Pineda E. Benítez-Páez A. (2016). Pangenome-wide and molecular evolution analyses of the *Pseudomonas aeruginosa* species. BMC Genomics 17, 1–14. doi: 10.1186/s12864-016-2364-4 26754847 PMC4710005

[B33] Mulani M. S. Kamble E. E. Kumkar S. N. Tawre M. S. Pardesi K. R. (2019). Emerging strategies to combat ESKAPE pathogens in the era of antimicrobial resistance: a review. Front. Microbiol. 10, 539. doi: 10.3389/fmicb.2019.00539 30988669 PMC6452778

[B34] Oliver A. Rojo-Molinero E. Arca-Suarez J. Beşli Y. Bogaerts P. Cantón R.. (2024). Pseudomonas aeruginosa antimicrobial susceptibility profiles, resistance mechanisms and international clonal lineages: update from ESGARS-ESCMID/ISARPAE Group. Clin. Microbiol. infection 30 (4), 469–480. doi: 10.1016/j.cmi.2023.12.026 38160753

[B35] Pan Y. P. Xu Y. H. Wang Z. X. Fang Y. P. Shen J. L. (2016). Overexpression of *MexAB-OprM* efflux pump in carbapenem-resistant Pseudomonas aeruginosa. Arch. Microbiol. 198, 565–571. doi: 10.1007/s00203-016-1215-7 27060003

[B36] Piddock L. J. (2006). Clinically relevant chromosomally encoded multidrug resistance efflux pumps in bacteria. Clin. Microbiol. Rev. 19, 382–402. doi: 10.1128/CMR.19.2.382-402.2006 16614254 PMC1471989

[B37] Qin S. Xiao W. Zhou C. Pu Q. Deng X. Lan L.. (2022). Pseudomonas aeruginosa: pathogenesis, virulence factors, antibiotic resistance, interaction with host, technological advances and emerging therapeutics. Signal transduction targeted Ther. 7, 199. doi: 10.1038/s41392-022-01056-1 PMC9233671 35752612

[B38] Rahbar M. Hamidi-Farahani R. Asgari A. Esmailkhani A. Soleiman-Meigooni S. (2021). Expression of RND efflux pumps mediated antibiotic resistance in Pseudomonas aeruginosa clinical strains. Microbial Pathogenesis 153, 104789. doi: 10.1016/j.micpath.2021.104789 33556480

[B39] Rakotovao-Ravahatra Z. D. Rahajamanana L. Rakotondraoelina L. Raskine L. Rasoanandrasana S. Rafalimanana C.. (2021). Comparison of bis NEG-D and API 20E for the identification of gram-negative bacilli in the laboratory of the university hospital of befelatanana antananarivo Madagascar. Eur. J. Biol. Biotechnol. 2, 76–80. doi: 10.24018/ejbio.2021.2.5.286

[B40] Recio R. Mancheño M. Viedma E. Villa J. Orellana M.Á. Lora-Tamayo J.. (2020). Predictors of mortality in bloodstream infections caused by Pseudomonas aeruginosa and impact of antimicrobial resistance and bacterial virulence. Antimicrobial Agents chemotherapy 64, 10–1128. doi: 10.1128/AAC.01759-19 PMC6985728 31767719

[B41] Reynolds D. Kollef M. (2021). The epidemiology and pathogenesis and treatment of P. aeruginosa infections: an update. Drugs 81, 2117–2131. doi: 10.1007/s40265-021-01635-6 34743315 PMC8572145

[B42] Richardson L. J. Rawlings N. D. Salazar G. A. Almeida A. Haft D. R. Ducq G.. (2019). Genome properties in 2019: a new companion database to InterPro for the inference of complete functional attributes. Nucleic Acids Res. 47, D564–D572. doi: 10.1093/nar/gky1013 30364992 PMC6323913

[B43] Robin X. Waterhouse A. M. Bienert S. Studer G. Alexander L. T. Tauriello G.. (2024). The SWISS-model repository of 3D protein structures and models. Open Access Database Datasets Drug Discov., 175–199. doi: 10.1002/9783527830497.ch6

[B44] Sanz-García F. Hernando-Amado S. Martínez J. L. (2018). Mutational evolution of *Pseudomonas aeruginosa* resistance to ribosome-targeting antibiotics. Front. Genet. 9, 451. doi: 10.3389/fgene.2018.00451 30405685 PMC6200844

[B45] Sennhauser G. Bukowska M. A. Briand C. Grütter M. G. (2009). Crystal structure of the multidrug exporter MexB from Pseudomonas aeruginosa. J. Mol. Biol. 389, 134–145. doi: 10.1016/j.jmb.2009.04.001 19361527

[B46] Sharma S. Devkota M. D. Pokhrel B. M. Banjara M. R. (2023). Detection of bla NDM– 1, mcr-1 and MexB in multidrug resistant *Pseudomonas aeruginosa* isolated from clinical specimens in a tertiary care hospital of Nepal. BMC Microbiol. 23, 153. doi: 10.1186/s12866-023-02906-w 37231387 PMC10210380

[B47] Swain S. S. Padhy R. N. (2016). Isolation of ESBL-producing gram-negative bacteria and in silico inhibition of ESBLs by flavonoids. J. Taibah Univ. Med. Sci. 11, 217–229. doi: 10.1016/j.jtumed.2016.03.007

[B48] Tsutsumi K. Yonehara R. Ishizaka-Ikeda E. Miyazaki N. Maeda S. Iwasaki K.. (2019). Structures of the wild-type MexAB–OprM tripartite pump reveal its complex formation and drug efflux mechanism. Nat. Commun. 10, 1520. doi: 10.1038/s41467-019-09463-9 30944318 PMC6447562

[B49] Tuon F. F. Dantas L. R. Suss P. H. Tasca Ribeiro V. S. (2022). Pathogenesis of the P. aeruginosa biofilm: a review. Pathogens 11, 300. doi: 10.3390/pathogens11030300 35335624 PMC8950561

[B50] Turnidge J. Abbott I. J. (2022). EUCAST breakpoint categories and the revised “I”: a stewardship opportunity for “I” mproving outcomes. Clin. Microbiol. Infection 28 (4), 475–476. doi: 10.1016/j.cmi.2021.12.007 34920117

[B51] Williams C. J. Headd J. J. Moriarty N. W. Prisant M. G. Videau L. L. Deis L. N.. (2018). MolProbity: more and better reference data for improved all-atom structure validation. Protein Sci. 27, 293–315. doi: 10.1002/pro.v27.1 29067766 PMC5734394

[B52] Yamasaki S. Zwama M. Yoneda T. Hayashi-Nishino M. Nishino K. (2023). Drug resistance and physiological roles of RND multidrug efflux pumps in Salmonella enterica, Escherichia coli and Pseudomonas aeruginosa. Microbiology 169, 001322. doi: 10.1099/mic.0.001322 37319001 PMC10333786

