# Supplementary material for: Decoding MexB efflux pump genes: structural, molecular, and phylogenetic analysis of multidrug-resistant and extensively drug-resistant Pseudomonas aeruginosa
Source: Front Cell Infect Microbiol. 2025 Jan 21;14:1519737. doi: 10.3389/fcimb.2024.1519737 (PMC11791646; doi:10.3389/fcimb.2024.1519737)
Supplement: Supplementary file 1 [file DataSheet1.docx]

**Table S1: Resistant status of isolates**

| **Isolates ID** | **Antibiotic classes and their agents** | | | | | | | | | | | | | |
| --- | --- | --- | --- | --- | --- | --- | --- | --- | --- | --- | --- | --- | --- | --- |
|  | **Penicillin’s** | **Cephalosporins** | | | **Carbapenems** | | **Aminoglycosides** | | **Tetracyclines** | **Fluoroquinolones** | | **Polymyxins** | | **Interpretation** |
|  | ***PIP-TAZ*** | ***CPZ*** | ***CTX*** | ***FEP*** | ***MEM*** | ***IPM*** | ***GEN*** | ***AK*** | ***TG*** | ***CIP*** | ***LEV*** | ***CT*** | ***PB*** |  |
| S1 | R | R | R | R | S | R | R | R | S | R | R | S | S | MDR |
| S2 | R | R | R | R | R | S | R | R | S | R | R | S | S | MDR |
| S3 | R | R | R | R | S | R | R | R | S | R | R | S | S | MDR |
| S4 | R | R | R | R | R | S | R | R | S | R | R | S | S | MDR |
| S5 | R | R | R | R | S | S | R | R | S | R | R | S | S | MDR |
| S6 | R | R | R | R | S | R | R | R | S | R | R | S | S | MDR |
| S7 | R | R | R | R | R | R | R | R | S | R | R | S | S | XDR |
| S8 | R | R | R | R | R | R | R | R | S | R | R | S | S | XDR |
| S9 | R | R | R | R | R | R | R | R | S | R | R | S | S | XDR |
| S10 | R | R | R | R | R | R | R | R | S | R | R | S | S | XDR |
| S11 | R | R | R | R | R | R | R | R | S | R | R | S | S | XDR |
| S12 | S | S | S | S | S | S | S | S | S | S | S | S | S | Sensitive |
| S13 | S | S | S | S | S | S | S | S | S | S | S | S | S | Sensitive |
| S14 | S | R | R | R | R | R | R | R | S | R | R | S | S | MDR |
| S15 | S | R | R | R | R | R | R | R | S | R | R | S | S | MDR |
| S16 | S | S | S | S | S | S | S | S | S | S | S | S | S | Sensitive |
| S17 | S | S | S | S | S | S | S | S | S | S | S | S | S | Sensitive |
| S18 | S | R | R | R | R | R | R | R | S | S | R | S | S | MDR |
| S19 | R | R | R | R | R | R | R | R | S | R | R | S | S | MDR |
| S20 | R | R | R | R | R | R | R | R | S | R | R | S | S | MDR |
| S21 | R | R | R | R | R | S | R | R | S | S | R | S | S | MDR |
| S22 | R | R | R | R | R | R | R | R | S | R | R | S | S | MDR |
| S23 | S | R | R | S | R | R | S | R | S | R | S | S | S | MDR |
| S24 | R | R | R | R | S | R | R | R | S | R | S | S | S | MDR |
| S25 | S | R | R | R | R | R | R | S | S | R | S | S | S | MDR |
| S26 | R | R | S | R | S | R | R | R | S | R | S | S | S | MDR |
| S27 | R | R | R | S | R | S | R | R | S | R | S | S | S | MDR |
| S28 | R | R | S | R | R | R | R | R | S | R | S | S | S | MDR |
| S29 | R | S | R | S | R | S | R | R | S | R | S | S | S | MDR |
| S30 | S | S | S | S | S | S | S | S | S | S | S | S | S | Sensitive |
| S31 | S | S | S | S | S | S | S | S | S | S | S | S | S | Sensitive |
| S32 | S | S | S | S | S | S | S | S | S | S | S | S | S | Sensitive |
| S33 | S | S | S | S | S | S | S | S | S | S | S | S | S | Sensitive |
| S34 | S | S | S | S | S | S | S | S | S | S | S | S | S | Sensitive |
| S35 | S | R | R | R | R | R | R | R | S | R | R | S | S | MDR |
| S36 | S | S | S | S | S | S | S | S | S | S | S | S | S | Sensitive |
| S37 | S | S | S | S | S | S | S | S | S | S | S | S | S | Sensitive |
| S38 | R | R | R | R | R | R | R | R | S | R | R | S | S | MDR |
| S39 | S | S | S | S | S | S | S | S | S | S | S | S | S | Sensitive |
| S40 | S | R | R | R | R | R | R | R | S | R | R | S | S | MDR |
| S41 | S | S | S | S | S | S | S | S | S | S | S | S | S | Sensitive |
| S42 | R | R | R | R | R | R | R | R | S | S | R | S | S | MDR |


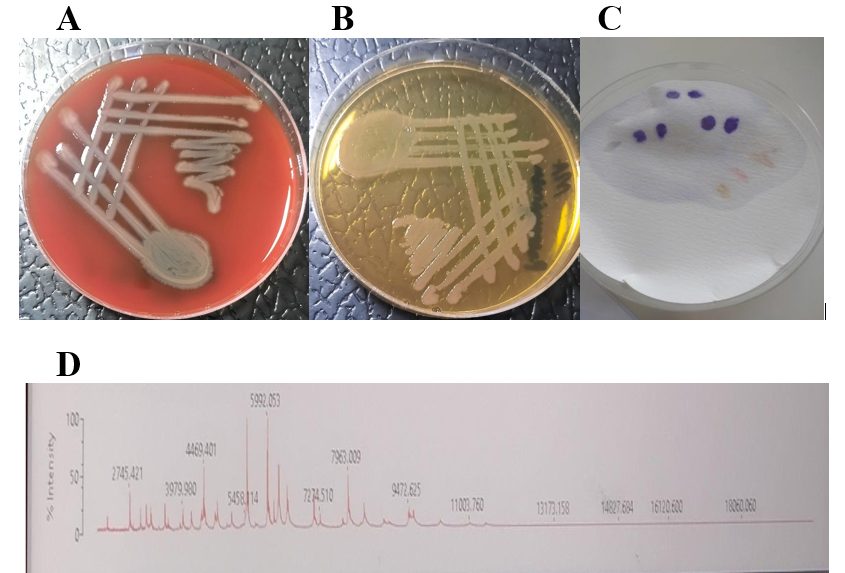


**Figure** **S1: “A”** showing large gray colored colonies, with irregular margins and metallic sheen on blood agar. **“B”** showing clear and non-lactose ferment colonies on MacConkey agar. **“C”** showing purple color on filter paper, this color produced when bacterial colonies was mixed with oxidase reagent indicated oxidase test positive (biochemical test) that is specific for *P. aeruginosa*. **“D”** MALDI-TOF analysis spectrum, the identified ions' mass-to-charge ratio (m/z), represented by the X-axis. Peaks are indicative of particular molecular weights of ionized proteins that are peculiar to the organism. The Y-axis shows the quantity or potency of the ions that were discovered. Small ribosomal proteins unique to *P. aeruginosa*, are represented by the wavelength range of 2705–2800 m/z. A crucial range for ribosomal subunit proteins is 5400–6000 m/z and proteins specific to *Pseudomonas* species are frequently found in peaks in this area. A peak in the 7241–7250 m/z region is frequently linked to *P. aeruginosa* and serves as a crucial identifying marker. 9000–11,000 m/z represents bigger metabolic or ribosomal proteins, specific to *Pseudomonas* species. During MALDI-TOF analysis, a distinct peak (~7241 m/z) corresponds with *P. aeruginosa* database entries.

**S1**

**
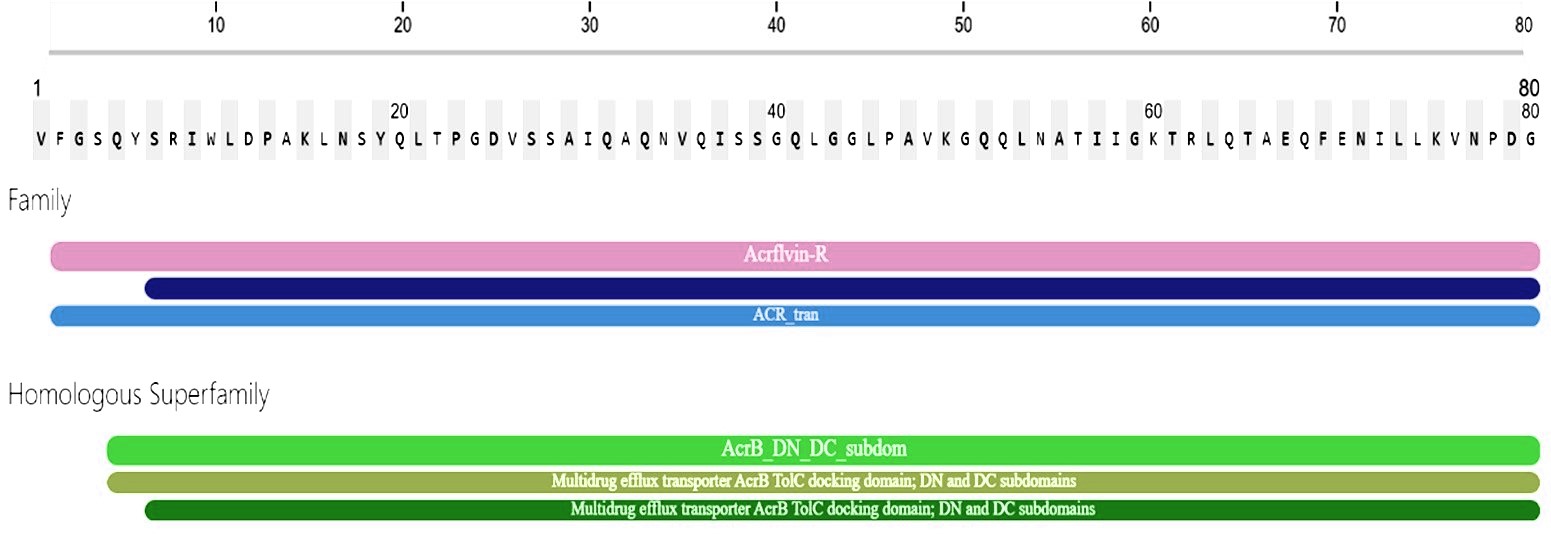
**

**S2**

**
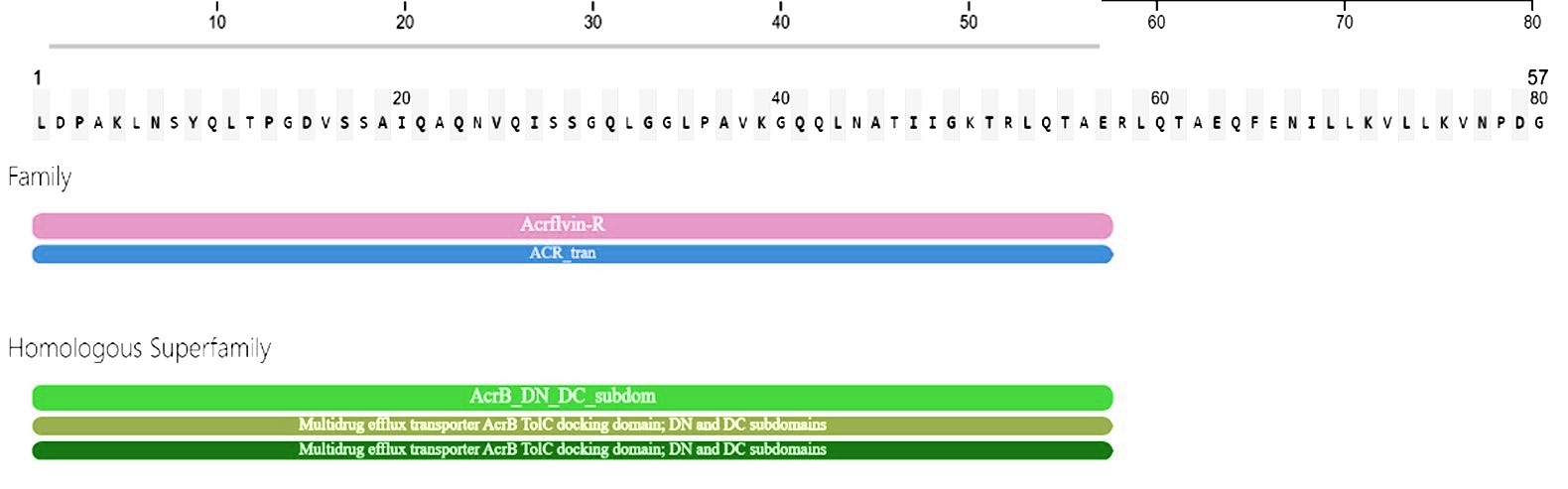
**

**S3**

**
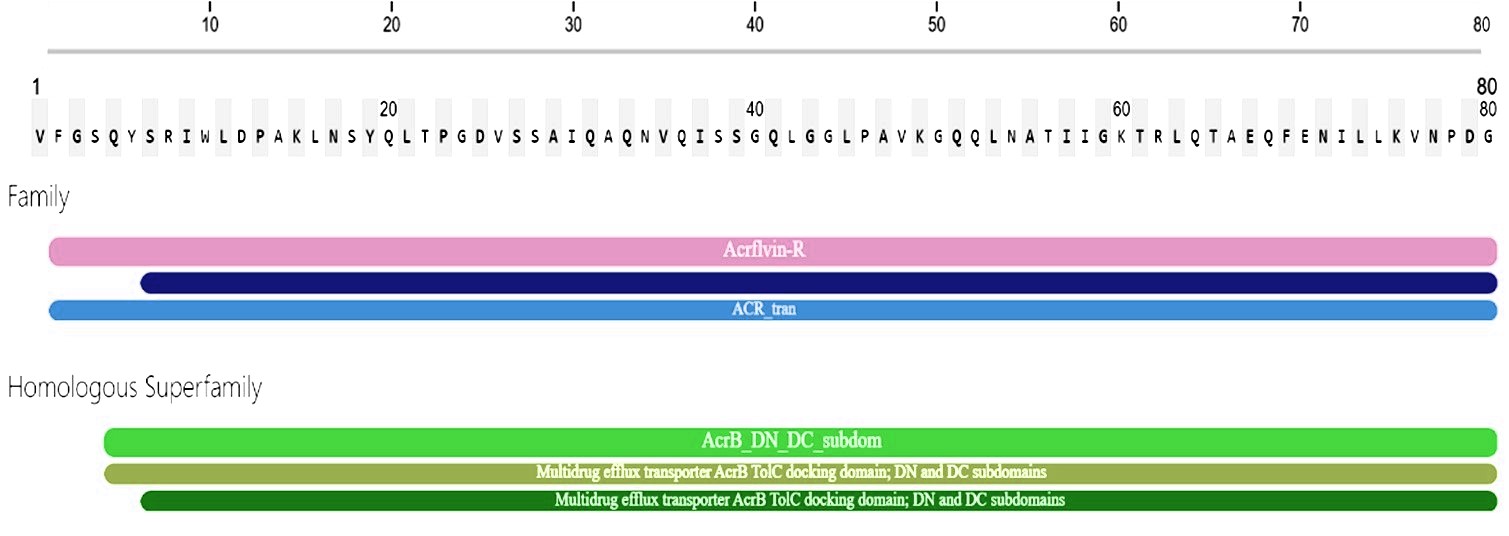
**

**S4**

**
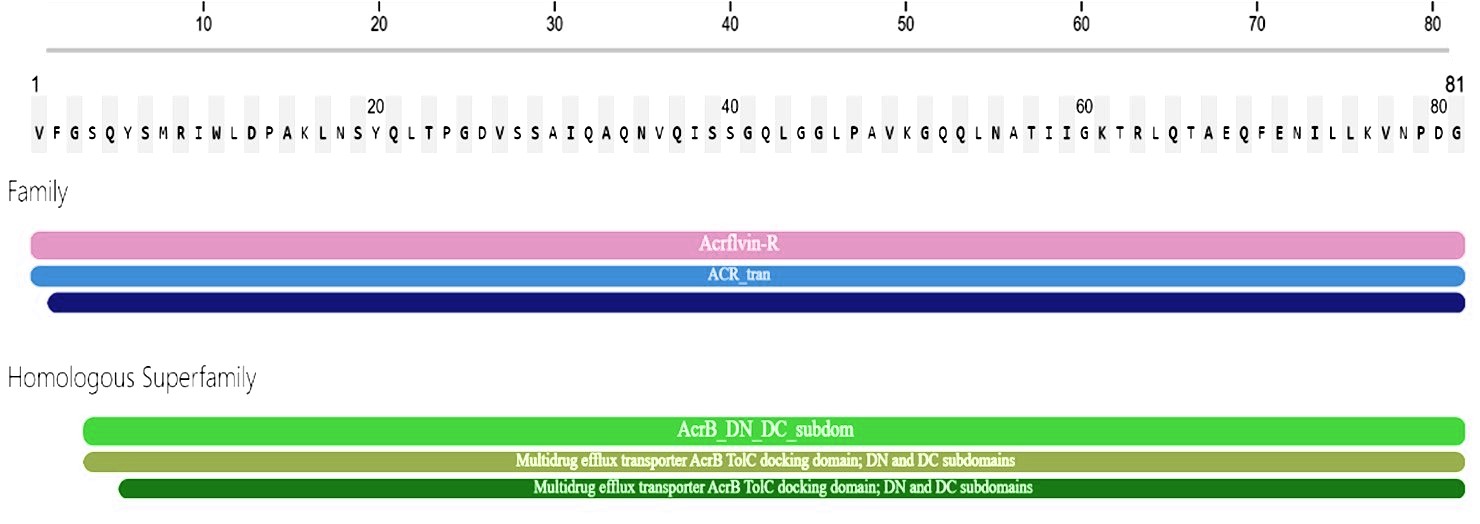
**

**S5**

**
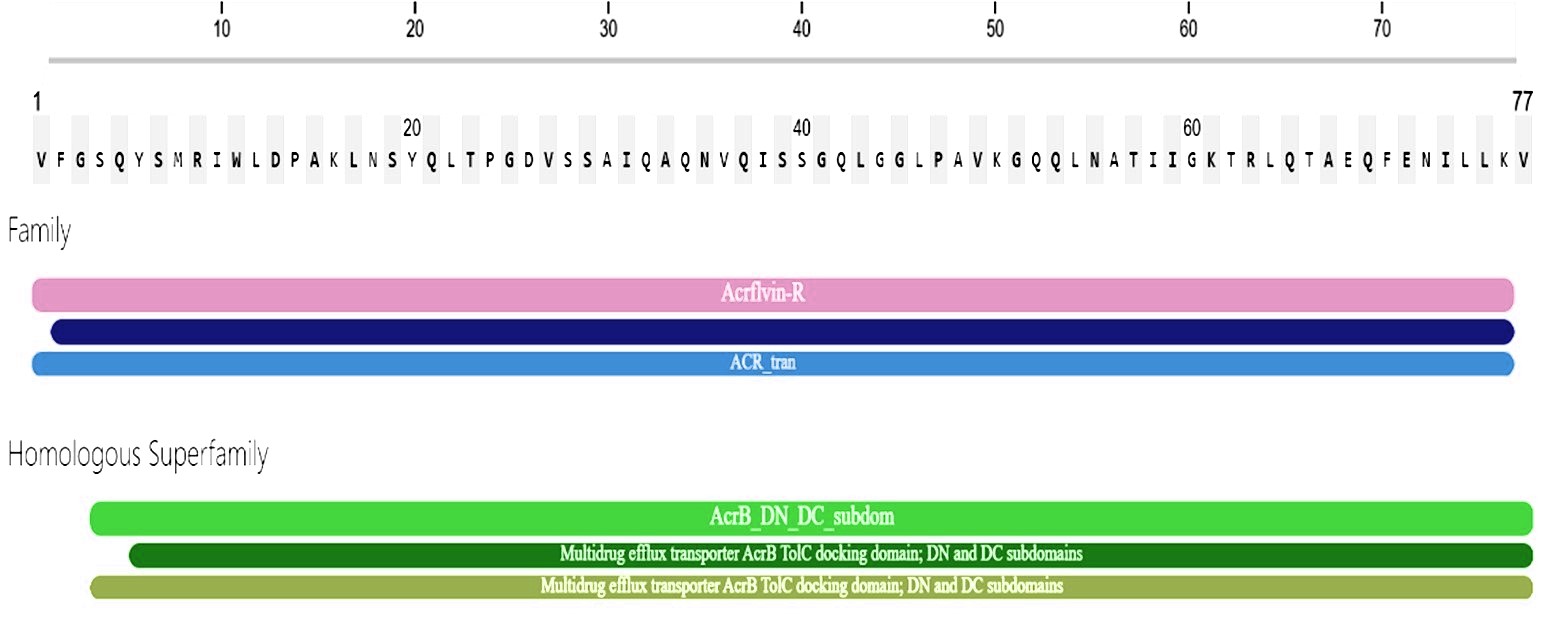
**

**S6**

**
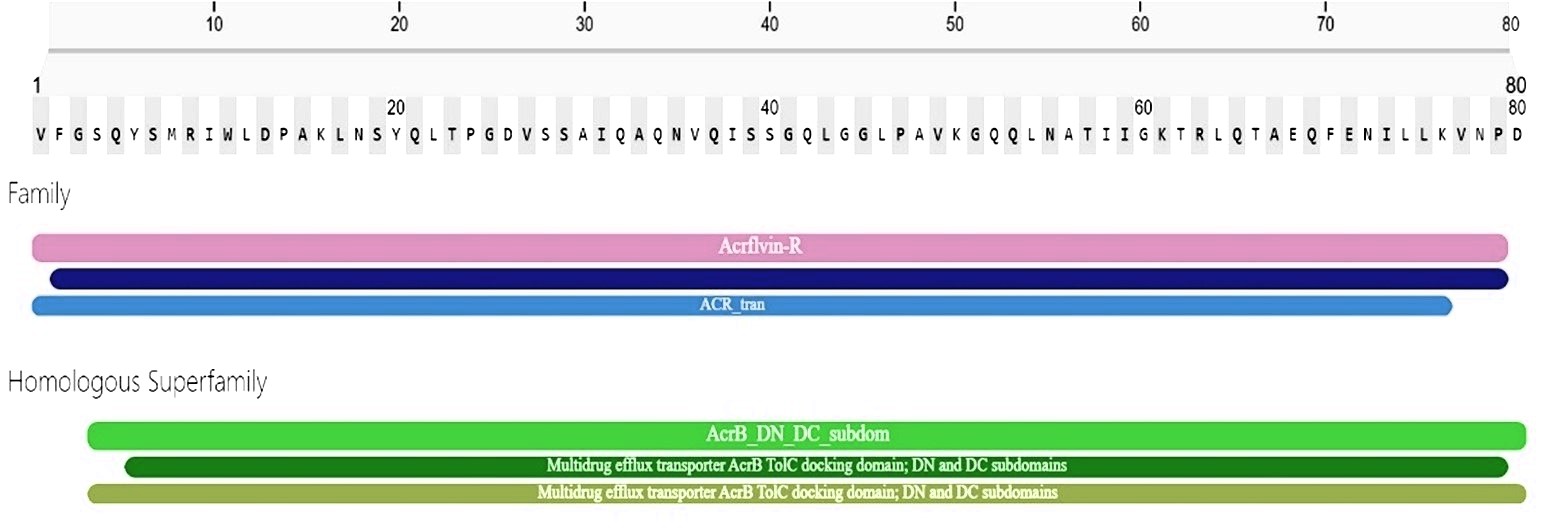
**

**S7**

**
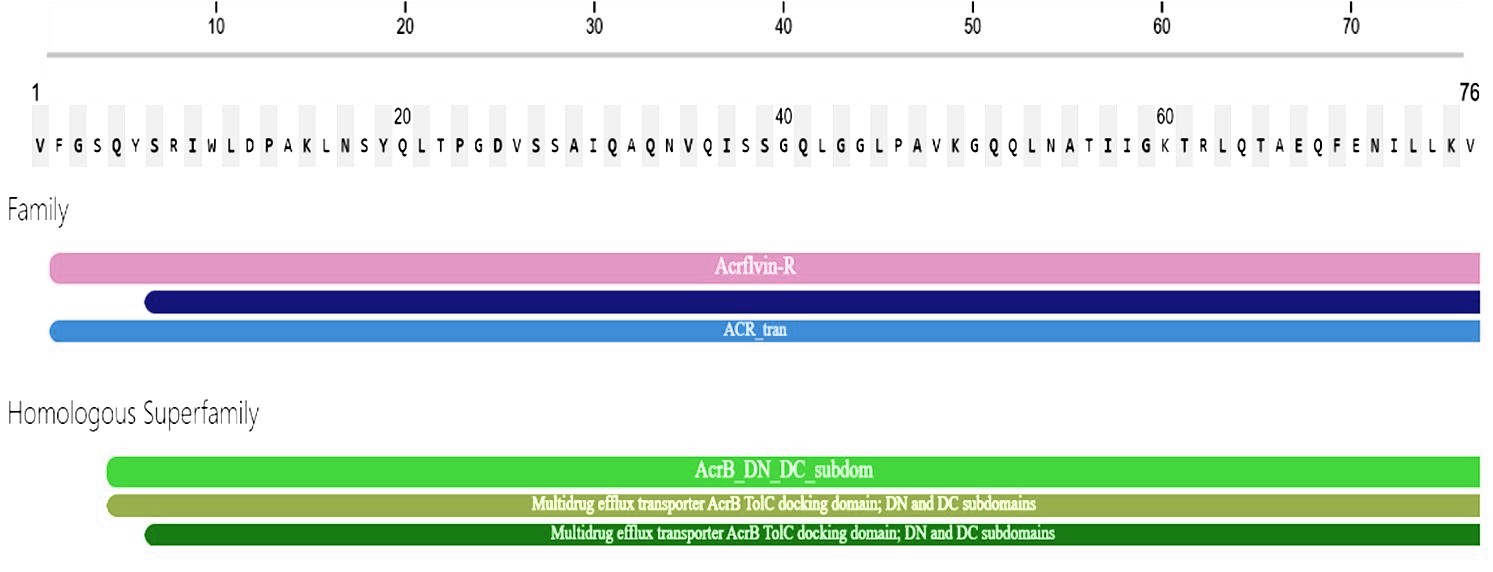
**

**S8**

**
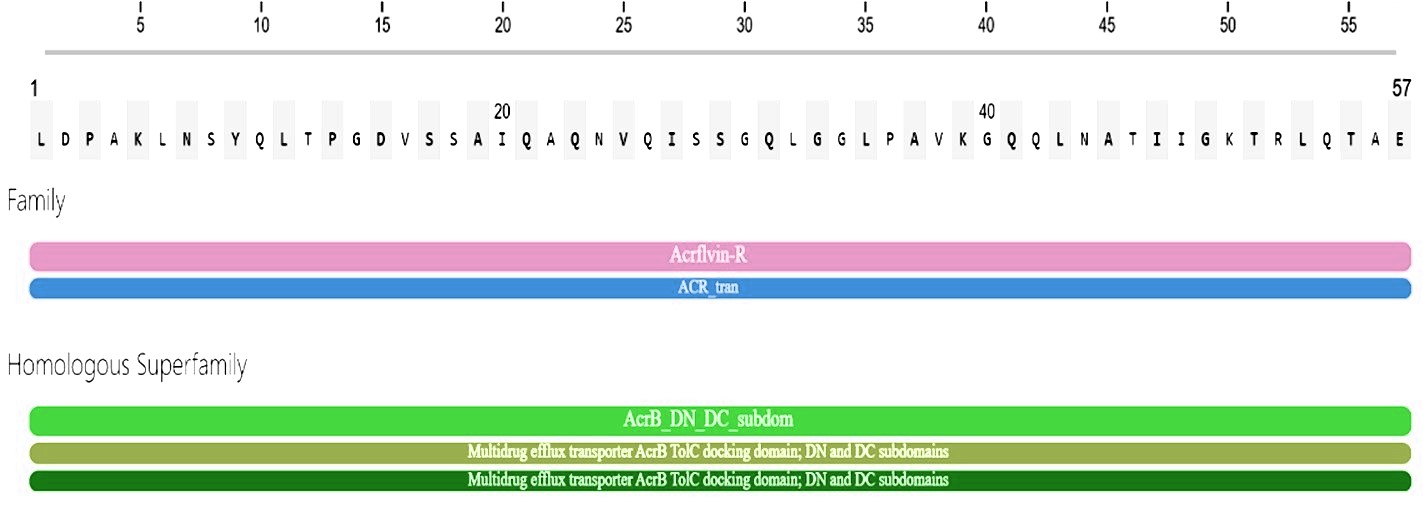
**

**S9**

**
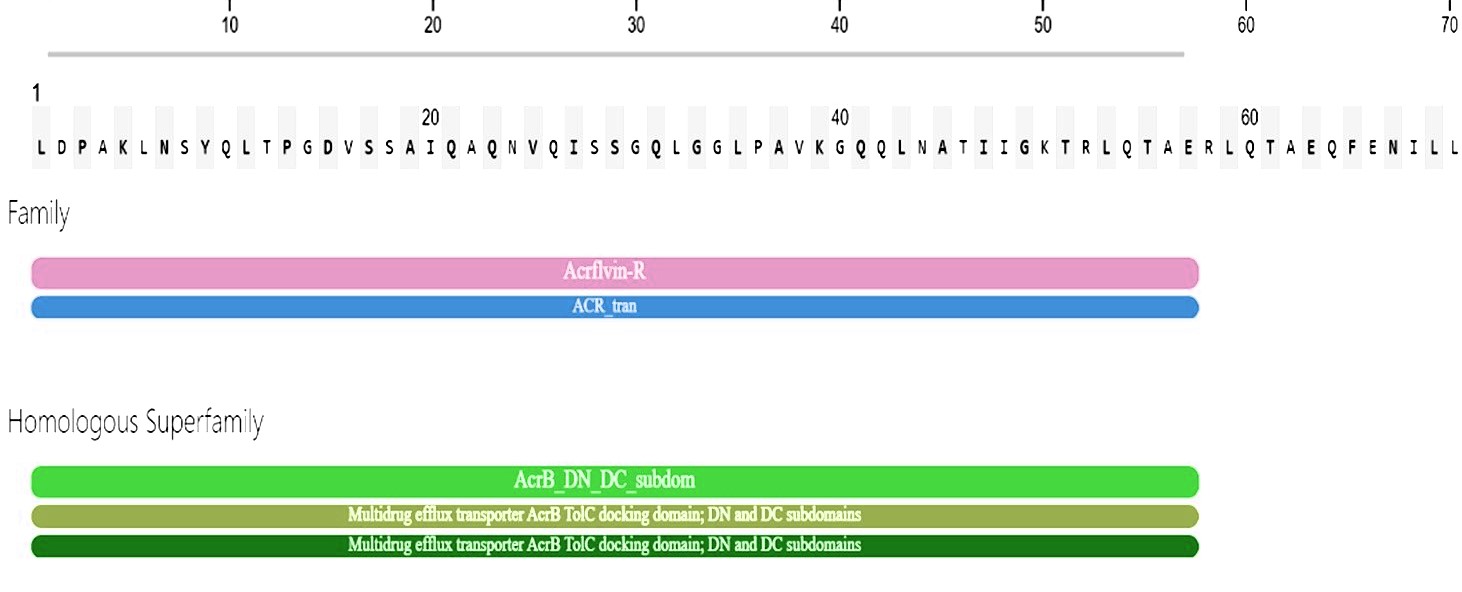
**

**S10**

**
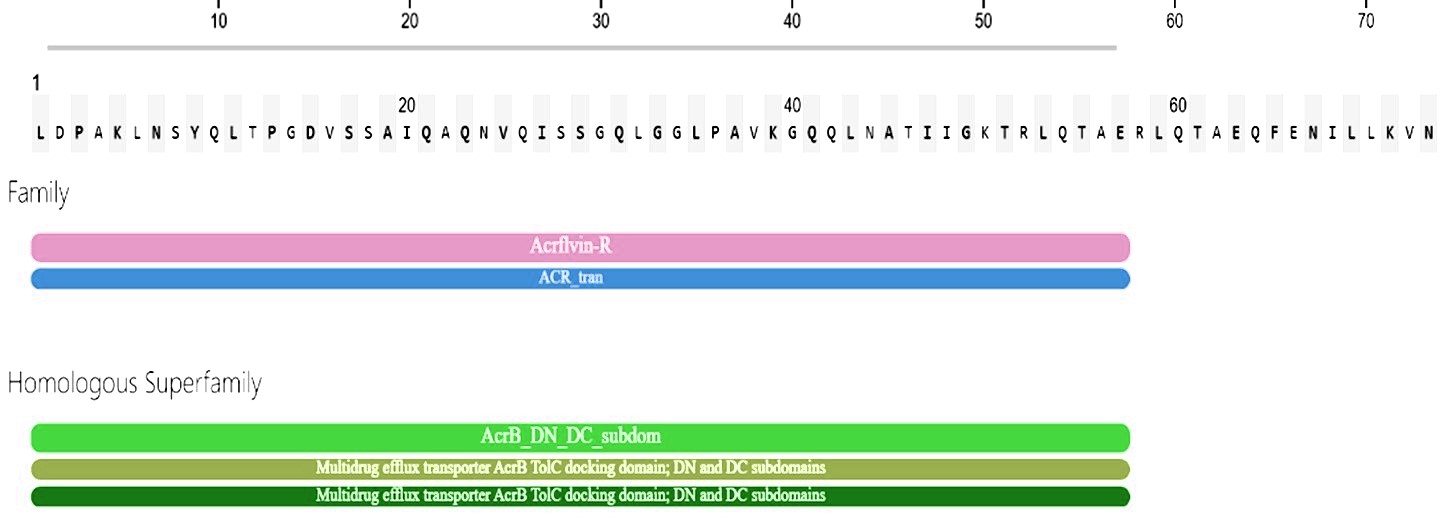
**

**S11**

**
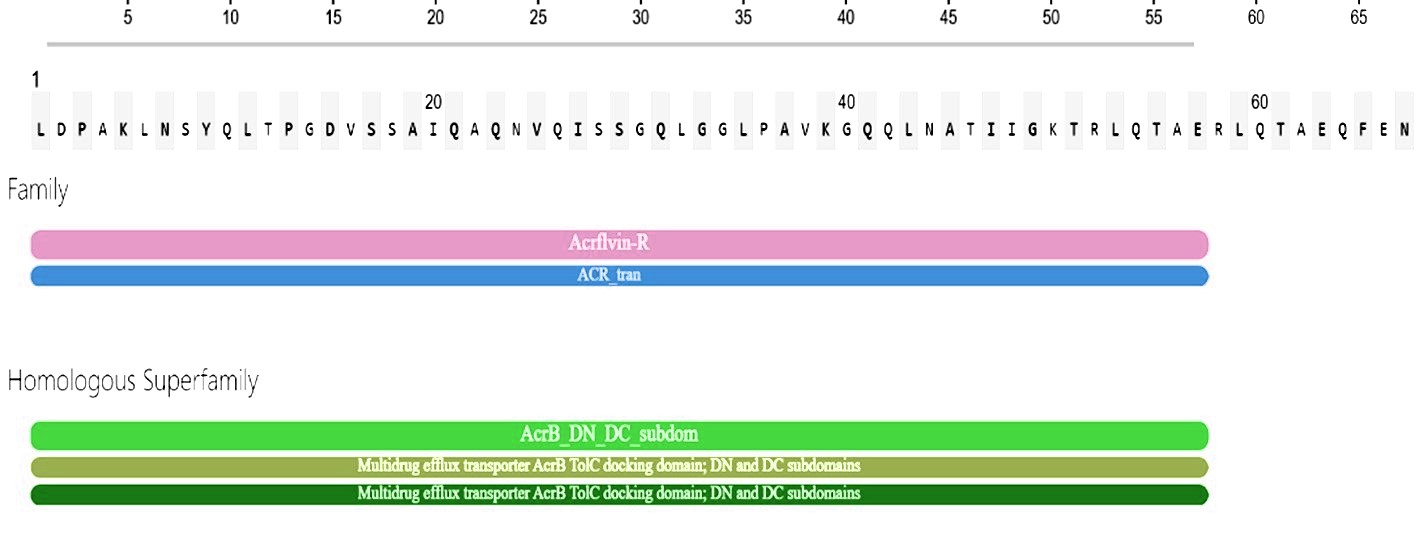
**

**Figure S2:** MDR S1-S6 and XDR S7-S11 Protein domain analysis by InterPro. At the top of the **images (S1-S11),** the sequence is displayed with positions numbered from **1 to 80**. Each position is labeled with the corresponding amino acid in the protein sequence (e.g., V, F, G, S, Q, L, D , P, P, A, K, etc.). The “**Family”** section identifies the **protein families** to which the sequence belongs. There are two main families highlighted: **Acriflavin-R (Pink Bar),** This refers to a family associated with the Acriflavin resistance protein (AcrR), part of the resistance-nodulation-cell division (RND) efflux pump proteins. It plays a role in **antibiotic resistance**, especially in efflux mechanisms that pump out toxic compounds from bacterial cells. **ACR_tran (Blue Bar):** refers to a **transporter family** associated with the AcrB protein, an essential component of the AcrAB-TolC efflux pump system. This transporter family is significant in drug resistance by actively exporting a wide variety of antibiotics out of the cell. The “**Homologous Superfamily”** section shows broader, evolutionary-related protein domains: **AcrB_DN_DC_subdom (Green Bar):** Indicates the **DN and DC subdomains** of the AcrB protein, specifically associated with the **TolC docking domain**. This domain is crucial for interacting with the outer membrane component **TolC** in the efflux pump complex. The label "**Multidrug efflux transporter AcrB TolC docking domains**" confirms the domain's involvement in drug export mechanisms.
